# A Proposed Probabilistic Extension of the Halpern and Pearl Definition of ‘Actual Cause’

**DOI:** 10.1093/bjps/axv056

**Published:** 2016-03-29

**Authors:** Luke Fenton-Glynn

**Affiliations:** Department of Philosophy University College London London, UK

## Abstract

Joseph Halpern and Judea Pearl ([2005]) draw upon structural equation models to develop an attractive analysis of ‘actual cause’. Their analysis is designed for the case of deterministic causation. I show that their account can be naturally extended to provide an elegant treatment of probabilistic causation.

1 Introduction
2 Preemption
3 Structural Equation Models
4 The Halpern and Pearl Definition of ‘Actual Cause’
5 Preemption Again
6 The Probabilistic Case
7 Probabilistic Causal Models
8 A Proposed Probabilistic Extension of Halpern and Pearl’s Definition
9 Twardy and Korb’s Account
10 Probabilistic Fizzling
11 Conclusion

## 1 Introduction

The investigation of actual (or ‘token’) causal relations—in addition to the investigation of generic (or ‘type’) causal relations—is an important part of scientific practice. For example, on various occasions in the history of science, paleontologists and geologists have been interested in determining the actual cause or causes of the extinction of the dinosaurs, cosmologists with the actual cause of the cosmic microwave background, astronomers with the actual causes of the perturbation of the orbit of Uranus and the perihelion precession of Mercury, and epidemiologists with the actual cause of the outbreak of the H7N9 avian influenza virus. Yet, despite the scientific importance of the discovery of actual causes, there remains a significant amount of philosophical work to be done before we have a satisfactory understanding of the nature of actual causation.

Halpern and Pearl ([2001], [2005]) have made progress on this front. They draw upon structural equation models (SEMs) to provide an innovative and attractive analysis (or ‘definition’, as they call it) of actual causation. Their analysis^1^ is closely related to analyses proposed by Pearl ([2009], Chapter 10),^2^ Hitchcock ([2001a], pp. 286–7, 289–90), and Woodward ([2005], pp. 74–86). Halpern and Pearl’s analysis handles certain cases that are counterexamples to these closely related accounts (see Pearl [2009], pp. 329–30; Weslake [forthcoming], Section 2), as well as handling many cases that pose problems for more traditional, non-structural equation-based analyses of actual causation (Halpern and Pearl [2001], pp. 197–202, [2005], pp. 856–69).

One limitation of Halpern and Pearl’s analysis and related accounts is that they are designed for the case of deterministic causation (see Halpern and Pearl [2005], p. 852; Hitchcock [2007], p. 498; Pearl [2009], p. 26). An extension of their analysis to enable it to handle probabilistic actual causation would be worthwhile, particularly in light of the probabilistic nature of many widely accepted scientific theories. In the following, I propose such an extension.

Before proceeding, it is worth noting that a refinement to Halpern and Pearl’s analysis has been proposed in (Halpern [2008], pp. 200–5; Halpern and Hitchcock [2010], pp. 389–94, 400–3, [2015], Section 6). The refined account preserves the core of Halpern and Pearl’s original analysis, but tweaks it slightly by strengthening one of its conditions so as to rule out certain alleged non-causes that are counted as actual causes by Halpern and Pearl’s analysis.^3^ However, doubt has been cast by Halpern ([unpublished], Section 1) and by Blanchard and Schaffer ([forthcoming], Section 3) upon whether this refinement to Halpern and Pearl’s original analysis is necessary (that is, whether the alleged counterexamples to Halpern and Pearl’s analysis are genuine).^4^ I will therefore take Halpern and Pearl’s analysis as my starting point in attempting to develop an analysis of actual causation adequate to the probabilistic case. As I shall explain in Section 5 below, if the proposed refinement to Halpern and Pearl’s analysis is necessary, it is plausible that it can be incorporated into my proposed analysis of probabilistic causation too.

The road map is as follows: In Section 2, I give an example of (deterministic) preemption, which poses problems for many traditional attempts to analyse actual causation in terms of counterfactuals (and, indeed, in terms of regularities and causal processes). In Section 3, I introduce the notion of an SEM. In Section 4, I outline Halpern and Pearl’s analysis of ‘actual cause’, which appeals to SEMs. In Section 5, I show that Halpern and Pearl’s analysis provides an attractive treatment of deterministic preemption. In Section 6, I describe an example of probabilistic preemption, which Halpern and Pearl’s analysis can’t (and wasn’t designed to) handle. In Section 7, I outline the notion of a probabilistic causal model. In Section 8, I draw upon the notion of a probabilistic causal model in proposing an extension of Halpern and Pearl’s analysis of ‘actual cause’ to the probabilistic case. I show that this extension yields an elegant treatment of probabilistic preemption. In Section 9, I outline an alternative attempt to extend analyses of actual causation in terms of SEMs to the probabilistic case, due to Twardy and Korb ([2011]). In Section 10, I show that Twardy and Korb’s proposal is subject to counterexamples which mine avoids. Section 11 concludes.

## 2 Preemption

Preemption makes trouble for attempts to analyse causation in terms of counterfactual dependence. Here’s an example.

PE: The New York Police Department is due to go on parade at the parade ground on Saturday. Knowing this, Don Corleone decides that when Saturday comes, he will order Sonny to go to the parade ground and shoot and kill Police Chief McCluskey. Not knowing Corleone’s plan, Don Barzini decides that when Saturday comes, he will order Turk to shoot and kill McCluskey. Turk is perfectly obedient and an impeccable shot; if he gets the chance, he will shoot and kill McCluskey. Nevertheless, Corleone’s headquarters are closer to the police parade ground than Barzini’s headquarters. If both Sonny and Turk receive their orders, then Sonny will arrive at the parade ground first, shooting and killing McCluskey before Turk gets the chance. Indeed, even if Sonny were to shoot and miss, McCluskey would be whisked away to safety before Turk had the chance to shoot. Sure enough, on Saturday, the dons order their respective minions to perform the assassination. Sonny arrives at the parade ground first, shooting and killing McCluskey before Turk arrives on the scene. Since Turk arrives too late, he does not shoot.

In this scenario, Corleone’s order is an actual cause of McCluskey’s death; Barzini’s order is not an actual cause, but merely a preempted backup. Still, McCluskey’s death doesn’t counterfactually depend upon Corleone’s order: if Corleone hadn’t issued his order and so Sonny hadn’t attempted to assassinate McCluskey, then Barzini would still have ordered Turk to shoot and kill McCluskey, and Turk would have obliged.

Such preemption cases pose a challenge for anyone attempting to analyse actual causation in terms of counterfactual dependence (see, for example, Lewis [1986a], pp. 200–2, [2004], pp. 81–2). Though I shall not attempt to show it here, they also pose a challenge for those seeking to analyse actual causation in terms of regularities (see Mackie [1965], p. 251; Lewis [1973a], p. 557; Strevens [2007], pp. 98–102; Baumgartner [2013], pp. 99–100; Paul and Hall [2013], pp. 74–5), and for those seeking to analyse actual causation in terms of causal processes (see Paul and Hall [2013], pp. 55–7, 77–8). Halpern and Pearl ([2001], [2005]) attempt to deal with such cases by appealing to SEMs.

## 3 Structural Equation Models

An SEM, M, is an ordered pair, 〈V,E〉, where V is a set of variables, and E is a set of structural equations.^5^ Each of the variables in V appears on the left-hand side of exactly one structural equation in E. The variables in V comprise two (disjoint) subsets: a set, U, of ‘exogenous’ variables, the values of which do not depend upon the values of any of the other variables in the model; and a set, Y, of ‘endogenous’ variables, the values of which do depend upon the values of other variables in the model. The structural equation for each endogenous variable, Y∈Y, expresses the value of *Y* as a function of other variables in V. That is, it has the form $Y=f_{Y}(V_{i},V_{j},V_{k},\ldots )$, where $V_{i},V_{j},V_{k},\ldots \in V\setminus Y$. Such a structural equation conveys information about how the value of *Y* counterfactually depends upon the values of the other variables in V.

Specifically, suppose that X,Z∈V and that $V\setminus X,Z=\{V_{1},V_{2},\ldots ,V_{n}\}$. Then, *X* appears as an argument in the function on the right-hand side of the structural equation for *Z* just in case there is a pair, $\left\{x^{\prime },x^{\prime \prime }\right\}$, of possible values of *X*; a pair, $\left\{z^{\prime },z^{\prime \prime }\right\}$, of possible values of *Z*; and a possible assignment of values, $V_{1}=v_{1},V_{2}=v_{2},\ldots ,V_{n}=v_{n}$ (abbreviated as $\vec{V}=\vec{v}$)^6^ to the variables in V∖X,Z such that it is true that (a) if it had been the case that $X=x^{\prime }$ and that $\vec{V}=\vec{v}$, then it would have been the case that $Z=z^{\prime }$; and (b) if it had been the case that $X=x^{\prime \prime }$ and that $\vec{V}=\vec{v}$, then it would have been the case that $Z=z^{\prime \prime }$. In other words, *X* appears on the right-hand side of the equation for *Z* just in case there is some assignment of values to the other variables in the model such that the value of *Z* depends upon that of *X* when the other variables take the assigned values (see Pearl [2009], p. 97; Hitchcock [2001a], pp. 280–1). If no variable appears on the right-hand side of the equation for *Z*, then *Z* is an exogenous variable. In that case, the structural equation for *Z* simply takes the form $Z=z^{*}$, where $z^{*}$ is the actual value of *Z*.

Any variables that appear as arguments in the function on the right-hand side of the equation for variable *V* are known as the ‘parents’ of *V*; *V* is a ‘child’ of theirs. The notion of an ‘ancestor’ is defined in terms of the transitive closure of parenthood, that of a ‘descendent’ in terms of the transitive closure of childhood.

Since structural equations encode information about counterfactual dependence, they differ from algebraic equations: given the asymmetric nature of counterfactual dependence, a structural equation $Y=f_{Y}(V_{i},V_{j},V_{k},\ldots )$ is not equivalent to $f_{Y}(V_{i},V_{j},V_{k},\ldots )=Y$ (see Pearl [1995], p. 672, [2009], pp. 27–9; Hitchcock [2001a], p. 280; Halpern and Pearl [2005], pp. 847–8; *inter alia*). Indeed, given a non-backtracking reading of counterfactuals (Lewis [1979], pp. 456–8), the counterfactuals entailed by $f_{Y}(V_{i},V_{j},V_{k},\ldots )=Y$ will typically be false where those entailed by $Y=f_{Y}(V_{i},V_{j},V_{k},\ldots )$ are true (see, for example, Hitchcock [2001a], p. 280; Halpern and Hitchcock [2015], p. 417). Limiting our attention to models entailing only non-backtracking counterfactuals helps to ensure that the SEMs that we consider possess the property of ‘acyclicity’: they are such that for no variable *V_i_* is it the case that the value of *V_i_* is a function of *V_j_*, which in turn is a function of *V_k_*, which is a function of …*V_i_*. Acyclic models entail a unique solution for each variable.

Analyses of actual causation in terms of SEMs typically appeal to only those models that encode only non-backtracking counterfactuals (Hitchcock [2001a], p. 280; Halpern and Hitchcock [2015], p. 417). Doing so is important if such analyses are to deliver the correct results about causal asymmetry. In virtue of their appeal to models encoding only non-backtracking counterfactuals, analyses of actual causation in terms of SEMs can be seen as continuous with the tradition, initiated by Lewis ([1973a]), of attempting to analyse causation in terms of such counterfactuals (see Hitchcock [2001a], pp. 273–4; Halpern and Pearl [2005], pp. 877–8).

An SEM, M=〈V,E〉, can be given a graphical representation by taking the variables in V as the nodes or vertices of the graph and drawing a directed edge (or ‘arrow’) from a variable *V_i_* to a variable *V_j_* ($V_{i},V_{j}\in V$), just in case *V_i_* is a parent of *V_j_* according to the structural equations in E. A ‘directed path’ can be defined as an ordered sequence of variables, $\langle V_{i},V_{j},\ldots ,V_{k}\rangle$, such that there is a directed edge from *V_i_* to *V_j_*, and a directed edge from *V_j_* to …*V_k_* (in other words, directed paths run from variables to their descendants).

In the terminology of Halpern and Pearl ([2005], pp. 851–2), where *y_i_* is a possible value of *Y_i_* and $Y_{i}\in Y$ (the set of endogenous variables), a formula of the form *Y_i_* = *y_i_* is a ‘primitive event’. In their notation, ϕ is a variable ranging over primitive events and Boolean combinations of primitive events (Halpern and Pearl [2005], p. 852).

One can evaluate a counterfactual of the form $V_{i}=v_{i}\wedge \ldots \wedge V_{k}=v_{k}□\to \phi$ with respect to an SEM, M=〈V,E〉, by replacing the equations for $V_{i},\ldots ,$ and *V_k_* in E with the equations $V_{i}=v_{i},\ldots ,$ and *V _k_* = *v_k_* (thus treating each of $V_{i},\ldots ,$ and *V_k_* as an exogenous variable), while leaving all other equations in E intact. The result is a new set of equations $E^{\prime }$. The counterfactual holds in the original model, M=〈V,E〉, just in case, in the solution to $E^{\prime },\;\phi$ holds. This gives us a method for evaluating, with respect to M, even those counterfactuals whose truth or falsity isn’t implied by any single equation in E considered alone (Hitchcock, [2001a], p. 283), for example, counterfactuals concerning how the value of a variable would differ if the values of its grandparents were different.

This ‘equation replacement’ method for evaluating counterfactuals models what would happen if the variables $V_{i},\ldots ,$ and *V_k_* were set to the values $V_{i}=v_{i},\ldots ,$ and *V_k_* = *v_k_* by means of ‘interventions’ (Woodward [2005], p. 98) or a small ‘miracles’ (Lewis [1979], p. 468).^7^ By replacing the normal equations for $V_{i},\ldots ,$ and *V_k_* (that is, the equations for these variables that appear in E) with the equations $V_{i}=v_{i},\ldots ,$ and *V_k_* = *v_k_*, while leaving all other equations intact, we are not allowing the values of $V_{i},\ldots ,$ and *V_k_* to be determined in the normal way, in accordance with their usual structural. Rather, we are taking them to be ‘miraculously’ set to the desired values (or at least set to the desired values via some process that is exogenous to the system being modelled, and which interferes with its usual workings; see Woodward [2005], p. 47). Evaluating counterfactuals in this way ensures the avoidance of backtracking (cf. Lewis [1979], pp. 456–8). Specifically, it ensures that we get the result that if $V_{i}=v_{i}\wedge \ldots \wedge V_{k}=v_{k}$, then the parents (and more generally, ancestors) of $V_{i},\ldots ,$ and *V_k_* would have had the same values (except where some of the variables $V_{i},\ldots ,$ and *V_k_* themselves have ancestors that are among $V_{i},\ldots ,$ and *V_k_*), while the children (and, more generally, descendants) of $V_{i},\ldots ,$ and *V_k_* are susceptible to change. This is because the structural equations for the ancestor and descendent variables (provided that they are not themselves among $V_{i},\ldots ,$ and *V_k_*) are left unchanged (cf. Pearl [2009], p. 205).

As observed by Halpern and Hitchcock ([2015], p. 420), there are at least two different views of the relationship between SEMs and counterfactuals to be found in the literature.^8^ One view—adopted by Hitchcock ([2001a], pp. 274, 279–84, 287) and Woodward ([2005], pp. 42–3, 110), *inter alia*—is that structural equations are just summaries of sets of (non-backtracking) counterfactuals: a structural equation of the form $Y=f_{Y}(V_{i},V_{j},V_{k},\ldots )$ simply summarizes a set of (non-backtracking) counterfactuals of the form $V_{i}=v_{i}\wedge V_{j}=v_{j}\wedge V_{k}=v_{k}\wedge \ldots □\to Y=y$, which, taken together, say what the value of *Y* would be for each possible assignment of values to $V_{i},V_{j},V_{k},\ldots$. More generally, on this view, an SEM, M, ‘encodes’ a set of counterfactuals—namely, the set of counterfactuals that are evaluated as true when the ‘equation replacement’ method is applied to M—which are given a non-backtracking semantics that is quite independent of M.

This independent semantics might be a broadly Lewisian semantics (Lewis [1979]), according to which a counterfactual $V_{i}=v_{i}\wedge V_{j}=v_{j}\wedge V_{k}=v_{k}\wedge \ldots □\to Y=y$ is true if *Y* = *y* holds in a world in which each of $V_{i},V_{j},V_{k},\ldots$ is set to the value specified in the antecedent by a ‘small miracle’.^9^ Alternatively, one might appeal to a Woodwardian semantics (Woodward [2005]), according to which the relevant world to consider is one in which each of $V_{i},V_{j},V_{k},\ldots$ is set to the specified value by an intervention.^10^ These accounts both avoid backtracking because on neither account are we to evaluate counterfactuals with reference to worlds in which their antecedents are realized as a result of different earlier conditions operating via the usual causal processes.

An alternative view of the relationship between structural equations and counterfactuals—adopted by Pearl ([2009], pp. 27–9, 33–8, 68–70, 202–15, 239–40)^11^—is that structural equations, rather than summarizing sets of counterfactuals, represent causal mechanisms, which are taken as primitives, and which are themselves taken to ground counterfactuals (see Halpern and Hitchcock [2015], p. 420). Pearl ([2009], p. 70), unlike Woodward,^12^ defines ‘interventions’ as ‘local surgeries’ (Pearl [2009], p. 223) on the causal mechanisms that he takes to be represented by structural equations. He takes such local surgeries to be formally represented by equation replacements (Pearl [2009], p. 70), and takes the equation replacement procedure to constitute a semantics for the sort of counterfactual conditional relevant to analysing actual causation (Pearl [2009], pp. 112–13, Chapter 7).^13^ As he puts it, this interpretation bases ‘the notion of interventions directly on causal mechanisms’ (Pearl [2009], p. 112), and takes ‘equation replacement’—which he construes as representing mechanism-modification—‘to provide a semantics for counterfactual statements’ (Pearl [2009], p. 113).

On the ‘primitive causal mechanisms’ view, the asymmetry of structural equations and the non-backtracking nature of the counterfactuals that (on this view) are given an ‘equation-replacement’ semantics follows from the asymmetry of the causal mechanisms themselves (cf. Pearl [1995], p. 672, [2009], pp. 27, 29, 69). Specifically, as Pearl notes, where mechanisms exhibit the desired causal asymmetry, the asymmetry of the equations representing those mechanisms (that is, the distinction between the dependent variable to appear on the left-hand side of the structural equation and the independent variables to appear on the right) can be ‘determined by appealing […] to the notion of hypothetical intervention and asking whether an external control over one variable in the mechanism necessarily affects the others’ (Pearl [2009], p. 228). Recalling that Pearl defines interventions in terms of local surgeries on mechanisms, the idea is that where an equation $Y=f_{Y}(V_{i},V_{j},V_{k},\ldots )$ represents an asymmetric causal mechanism, the value of *Y* would change under local surgeries on the mechanism that affect the values of *V_i_*, *V_j_*, *V_k_*, …, but the values of *V_i_*, *V_j_*, *V_k_*, … would not change under local surgeries that affect the value of *Y*.

For present purposes, there is no need to choose between the ‘summaries of counterfactuals’ and ‘primitive causal mechanisms’ construals of structural equations. It is worth noting, however, that the choice between the two approaches may have implications for the potential reductivity of an analysis of actual causation in terms of SEMs. If SEMs represent sets of primitive causal mechanisms, then an analysis of actual causation in terms of SEMs will not reduce actual causation to non-causal facts. By contrast, on the ‘summaries of counterfactuals’ construal, an analysis of actual causation in terms of SEMs will potentially be reductive if the counterfactuals summarized can be given a semantics—perhaps along the lines of (Lewis [1979])—that doesn’t appeal to causal facts. Reduction will not, however, be achieved if one instead adopts a semantics that appeals to causal notions, such as Woodward’s ‘interventionist’ semantics (see Woodward [2005], p. 98).

Nevertheless, even if the analysis is non-reductive, it is plausible that it might still be illuminating. Woodward ([2005], pp. 104–7) has rather convincingly argued that, although non-reductive, an analysis of causation in terms of SEMs that summarize counterfactuals that are given by his interventionist semantics can be illuminating and can avoid viciously circularity.^14^ Meanwhile, Halpern and Hitchcock ([2015], p. 420) argue that if we adopt the primitive causal mechanisms construal of structural equations, we can still give an illuminating (though non-reductive) analysis of actual causation in terms of SEMs. In particular, they observe that—on this construal—SEMs themselves ‘do not directly represent relations of actual causation’, but merely an ‘underlying “causal structure”’ (Halpern and Hitchcock [2015], p. 420) in terms of which actual causal relations can be understood. A similar view appears to be taken by Pearl ([2009]). On Pearl’s view, such an analysis reduces actual causation to facts about ‘causal mechanisms’ (Pearl [2009], p. 112), which are construed as ‘invariant linkages’ (Pearl [2009], p. 223) or stable, law-like relationships (Pearl [2009], pp. 224–5, 239), which are not themselves to be analysed in terms of actual causation (cf. Halpern and Pearl [2005], p. 849).

I shall not argue here that Halpern and Pearl’s definition of actual causation, or the probabilistic extension that I shall propose in Section 8, can be converted into a fully reductive analysis of actual causation in non-causal terms. I agree with the authors just cited that an analysis can be illuminating without being fully reductive.

## 4 The Halpern and Pearl Definition of ‘Actual Cause’

Before stating Halpern and Pearl’s analysis of actual causation, it is necessary to introduce some more of their terminology. Recall that, given an SEM, M=〈V,E〉, Halpern and Pearl ([2005], pp. 851–2) call a formula of the form *Y* = *y* a primitive event, where Y∈Y (Y being the subset of V that comprises the endogenous variables) and *y* is a possible value of *Y*. They take ϕ to be a variable ranging over primitive events and Boolean combinations of primitive events (Halpern and Pearl [2005], p. 852).

Where $Y_{1},\ldots ,Y_{n}$ are variables in Y (each of which is distinct from any variable that appears in the formula ϕ), Halpern and Pearl ([2005], p. 852) call a formula of the form $[Y_{1}=y_{1},\ldots ,Y_{n}=y_{n}]\phi$, which they abbreviate $[\vec{Y}=\vec{y}]\phi$, a ‘basic causal formula’. Such a formula says that if it had been the case that $Y_{1}=y_{1},\ldots$ and *Y_n_* = *y_n_*, then it would have been the case that ϕ (Halpern and Pearl [2005], p. 852). As such $[\vec{Y}=\vec{y}]\phi$ is simply a notational variant on $Y_{1}=y_{1}\wedge \ldots \wedge Y_{n}=y_{n}□\to \phi$ (Pearl [2009], pp. 70, 108; cf. Halpern and Pearl [2005], p. 852).^15^ Finally, a ‘context’ is an assignment of values to the variables in U (that is, the exogenous variables in V) (Halpern and Pearl [2005], p. 849). That is, where $U=\{U_{1},\ldots ,U_{m}\}$, a context is an assignment of a value to each *U_i_*: $U_{1}=u_{1},\ldots ,U_{m}=u_{m}$. Such an assignment is abbreviated to $\vec{U}=\vec{u}$ or simply as $\vec{u}$ (Halpern and Pearl [2005], pp. 847, 849).

Given context $\vec{U}=\vec{u}$, the structural equations for the endogenous variables Y in acyclic SEM, M, determine a unique value for each of the variables in Y. Halpern and Pearl ([2005], p. 852) write $(M,\vec{u})⊨\phi$ if ϕ holds in the unique solution to the model $M^{\prime }$ that results from M when the equations in M for the exogenous variables U are replaced with equations setting these variables to the values that they are assigned in the context $\vec{U}=\vec{u}$. That is, $(M,\vec{u})⊨\phi$ says that if the exogenous variables in M were to take the values $\vec{U}=\vec{u}$, then (according to M) ϕ would hold. Moreover, Halpern and Pearl ([2005], p. 852) write that $(M,\vec{u})⊨[\vec{Y}=\vec{y}]\phi$ if ϕ holds in the unique solution to the model $M^{\prime \prime }$ that results from $M^{\prime }$ by replacing the equations for the variables $\vec{Y}$ with equations setting these variables equal to the values $\vec{Y}=\vec{y}$. That is, $(M,\vec{u})⊨[\vec{Y}=\vec{y}]\phi$ says that given context $\vec{U}=\vec{u}$, the causal formula—that is, counterfactual—$[\vec{Y}=\vec{y}]\phi$ holds (according to M). By contrast, $(M,\vec{u})⊭[\vec{Y}=\vec{y}]\phi$ says that given context $\vec{U}=\vec{u}$, the causal formula $[\vec{Y}=\vec{y}]\phi$ does not hold (according to M). Similarly, $(M,\vec{u})⊭\phi$ says that, in the context $\vec{U}=\vec{u},\;\phi$ does not hold (according to M).

The types of events that Halpern and Pearl allow to be actual causes are primitive events and conjunctions of primitive events (for simplicity, I’ll take a primitive event to be a limiting case of a conjunction of primitive events in what follows). That is, actual causes have the form $X_{1}=x_{1}\wedge \ldots \wedge X_{n}=x_{n}$ (for $X_{1},\ldots ,X_{n}\in Y$), abbreviated as $\vec{X}=\vec{x}$ (Halpern and Pearl [2001], p. 196, [2005], p. 853). The events that they allow as effects are primitive events and arbitrary Boolean combinations of primitive events (Halpern and Pearl [2001], p. 196; [2005], p. 853). They define actual cause as follows (Halpern and Pearl [2001], pp. 196–7).^16^^,^^17^^,^^18^^,^^19^

AC: $\vec{X}=\vec{x}$ is an actual cause *of*ϕ*in*$\left(M,\vec{u}\right)$ [[that is, in model M given the context $\vec{u}$]] if the following three conditions hold:

AC1. $(M,\vec{u})⊨(\vec{X}=\vec{x})\wedge \phi$ (that is, both $\vec{X}=\vec{x}$ and ϕ are true in the actual world).
AC2. There exists a partition $\left(\vec{Z},\vec{W}\right)$ of Y [[that is, the set of endogenous variables in the model M]] with $\vec{X}\subseteq \vec{Z}$ and some setting $\left(\vec{x}\prime ,\vec{w}\prime \right)$ of the variables in $\left(\vec{X},\vec{W}\right)$ such that [[where]] $(M,\vec{u})⊨Z_{i}=z_{i}^{*}$ for [[all]] $Z_{i}\in \vec{Z}$, [[the following holds:]]
  - $(M,\vec{u})⊨[\vec{X}=\vec{x}\prime ,\vec{W}=\vec{w}\prime ]\neg \phi$. In words, changing $\left(\vec{X},\vec{W}\right)$ from $\left(\vec{x},\vec{w}\right)$ to $\left(\vec{x}\prime ,\vec{w}\prime \right)$ changes ϕ from true to false
  - $(M,\vec{u})⊨[\vec{X}=\vec{x},\vec{W}=\vec{w}\prime ,\vec{Z}\prime ={\vec{z}}^{*}]\phi$ for all subsets $\vec{Z}\prime$ of $\vec{Z}$. In words, setting $\vec{W}$ to $\vec{w}\prime$ should have no effect on ϕ, as long as $\vec{X}$ is kept at its [[actual]] value $\vec{x}$, even if all the variables in an arbitrary subset of $\vec{Z}$ are set to their original values in the context $\vec{u}$.
AC3. $\vec{X}$ is minimal; no [[strict]] subset of $\vec{X}$ satisfies conditions AC1 and AC2. Minimality ensures that only those elements of the conjunction $\vec{X}=\vec{x}$ that are essential for changing ϕ in AC2(a) are considered part of a cause; inessential elements are pruned.

As Halpern and Pearl ([2001], p. 197) observe, the core of the definition is AC2. They observe that, informally, the variables in $\vec{Z}$ can be thought of as describing the ‘active causal process’ from $\vec{X}=\vec{x}$ to ϕ (Halpern and Pearl [2001], p. 197).^20^ They demonstrate (Halpern and Pearl [2005], pp. 879–80) that where a partition ($\vec{Z},\vec{W}$) is such that AC2 is satisfied, all variables in $\vec{Z}$ lie on a directed path from a variable in $\vec{X}$ to a variable in ϕ. The variables in $\vec{W}$, on the other hand, are not part of the active causal process (Halpern and Pearl [2005], p. 854).

Condition AC2(a) says that there exists a (non-actual) assignment $\vec{X}=\vec{x}\prime$ of possible values to the variables $\vec{X}$ such that if the variables $\vec{X}$ had taken the values $\vec{X}=\vec{x}\prime$, while the variables $\vec{W}$ had taken the values $\vec{W}=\vec{w}\prime$, then ¬ϕ would have held (Halpern and Pearl [2005], p. 854). Condition AC2(a) thus doesn’t require that ϕ straightforwardly counterfactually depends upon $\vec{X}=\vec{x}$ but rather requires (more weakly) that ϕ counterfactually depends upon $\vec{X}=\vec{x}$ under the contingency (that is, when it is built into the antecedent of the counterfactual) that $\vec{W}=\vec{w}\prime$ (Halpern and Pearl [2005], p. 854).

On the other hand, condition AC2(b) is designed to ensure that it is $\vec{X}=\vec{x}$, operating via the directed path(s) upon which the variables in $\vec{Z}$ lie, rather than $\vec{W}=\vec{w}$, that is causally responsible for ϕ. It does this by requiring that if the variables in $\vec{X}$ had taken the values $\vec{X}=\vec{x}$, and any arbitrary subset $\vec{Z}\prime$ of $\vec{Z}$ had taken their actual values $\vec{Z}\prime ={\vec{z}}^{*}$ while the values of the variables in $\vec{W}$ had taken the values $\vec{W}=\vec{w}\prime$, then ϕ would still have held (Halpern and Pearl [2005], pp. 854–5).

Halpern and Pearl’s definition AC relativizes the notion of actual causation to an SEM. This might be thought a slightly odd feature, since ordinarily we take actual causation to be an objective feature of the world that is not model-relative. Others who have attempted to analyse actual causation in terms of SEMs have sought to avoid model-relativity by suggesting that $\vec{X}=\vec{x}$ is an actual cause of ϕ*simpliciter*, provided that there exists at least one ‘appropriate’ SEM relative to which $\vec{X}=\vec{x}$ satisfies the criteria for being a (model-relative) actual cause of ϕ (Hitchcock [2001a], p. 287; cf. Woodward [2008], p. 209).^21^ We could use this strategy to extract a non-model-relative notion of actual causation from Halpern and Pearl’s definition. Of course, this strategy requires us to say what constitutes an appropriate SEM. Though this isn’t an altogether straightforward task, progress has been made (see Hitchcock [2001a], p. 287; Halpern and Hitchcock [2010], pp. 394–9; Blanchard and Schaffer [forthcoming], Section 1). I won’t review all of the criteria for model appropriateness that have been advanced in the literature; suffice it to say that the SEMs outlined below satisfy all of the standard criteria that have been suggested.

One criterion is worth mentioning, however. Hitchcock has suggested that, to be appropriate, a model, M, must ‘entail no false counterfactuals’ (Hitchcock [2001a], p. 287). By this he means that evaluating counterfactuals with respect to M by means of the equation replacement method doesn’t lead to evaluations of counterfactuals as true when they are, in fact, false (Hitchcock [2001a], p. 283).^22^ I shall discuss an analogous criterion for the appropriateness of probabilistic causal models when I discuss the latter in Section 7 below.

## 5 Preemption Again

To see that Halpern and Pearl’s definition AC delivers the correct result in the simple preemption case described in Section 2 above, it is necessary to provide an SEM. I will call the model developed in this section ‘PE’.

Let *C*, *B*, *S*, *T*, and *D* be binary variables, where *C* takes a value of one if Corleone orders Sonny to shoot and kill McCluskey, and takes a value of zero if he doesn’t; *B* takes a value of one if Barzini orders Turk to shoot and kill McCluskey, zero if he doesn’t; *S* takes a value of one if Sonny shoots, zero if he doesn’t; *T* takes a value of one if Turk shoots, zero if he doesn’t; and *D* takes a value of one if McCluskey dies, and zero if he survives. To these variables, let us add two more binary variables: *CI*, which takes a value of one if Corleone intends to issue his order and zero if he doesn’t; and *BI*, which takes a value of one if Barzini intends to issue his order, and zero if he doesn’t.^23^

The system of structural equations for this example is as follows:

- *CI* = 1;
- *BI* = 1;
- *C* = *CI*;
- *B* = *BI*;
- *S* = *C*;
- T=Min{B,1−S};
- D=Max{S,T}.

In our model PE, U={CI,BI}. That is, the variables *CI* and *BI* are the exogenous variables. Equations (i) and (ii) simply state their actual values, *CI* = 1 and *BI* = 1, representing the fact that Corleone forms his intention and that Barzini forms his intention. Thus, the actual context is $\vec{u}=\{CI=1,BI=1\}$.

In PE, Y={C,B,S,T,D}. That is, the variables *C*, *B*, *S*, *T*, and *D* are the endogenous variables. The structural equations for these variables express their values as a function of other variables in the model. Equation (iii) says that if Corleone had formed the intention to issue his order, then he would have issued it, but that he wouldn’t have issued it if he hadn’t formed the intention to do so. We might express this informally by saying that Corleone issues his order just in case he forms the intention to. Equation (iv) then says that Barzini issues his order just in case he forms the intention to; Equation (v) says that Sonny shoots just in case Corleone issues his order; Equation (vi) says that Turk shoots just in case Barzini issues his order and Sonny does not shoot; and Equation (vii) says that McCluskey dies just in case either Sonny or Turk shoots.

Given the context, $\vec{u}=\{CI=1,BI=1\}$, the values of the (endogenous) variables in Y are uniquely determined in accordance with the structural equations. The unique solution to our set of structural equations is: *CI* = 1, *BI* = 1, *C* = 1, *B* = 1, *S* = 1, *T* = 0, *D* = 1. That is, Corleone forms the intention to issue his order; Barzini forms the intention to issue his order; Corleone issues his order; Barzini issues his order; Sonny shoots; Turk doesn’t shoot; McCluskey dies.

**Figure 1.**
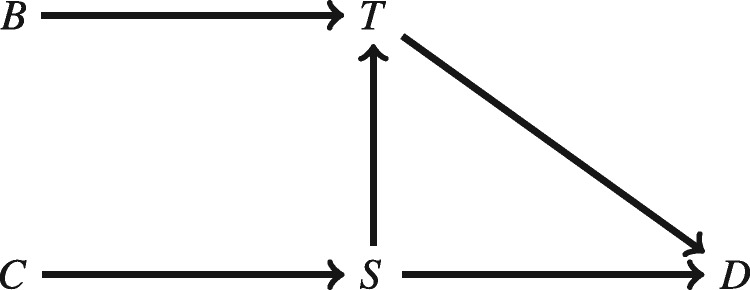
A graphical representation of the model PE.

We can give PE a graphical representation by following the conventions for drawing such graphs that were outlined in Section 3. Following Halpern and Pearl ([2005], p. 862), I omit exogenous variables from the graph. The resulting graph is given as Figure 1.

With the model PE of our preemption case in hand, we are in a position to see that AC correctly diagnoses Corleone’s order (*C* = 1) as an actual cause of McCluskey’s death (*D* = 1). To see that it does, let $\vec{X}=\{C\}$, with $\vec{x}=\{C=1\}$ and $\vec{x}\prime =\{C=0\}$. Let ϕ be *D* = 1. In the solution to the structural equations, given the actual context, $\vec{u}=\{CI=1,BI=1\}$, *C* = 1 and *D* = 1 hold. So condition AC1 of AC is satisfied. Condition AC3 is also satisfied, since $\vec{X}=\{C\}$ has no (non-empty) strict subsets. So everything hinges on whether AC2 is satisfied.

To see that AC2 is satisfied, let $\vec{Z}=\langle C,S,D\rangle$, let $\vec{W}=\langle B,T\rangle$, and let $\vec{w}\prime =\{B=1,T=0\}$. First note that AC2(a) is satisfied because in the set of structural equations that results from replacing Equation (iii) with Equation (iii′) *C* = 0, and the Equations (iv) and (vi) with Equation (iv′) *B* = 1 and Equation (vi′) *T* = 0, the solution for *D* is *D* = 0. This means that it is true that

(PE,{CI=1,BI=1})⊨[C=0∧B=1∧T=0]¬D=1.

That is, in the model PE and the context {CI=1;BI=1}, it is true that if Corleone hadn’t issued his order and Barzini had issued his order but Turk hadn’t shot, then McCluskey wouldn’t have died.

To see that AC2(b) is satisfied, note that the structural equations in PE ensure that if *C* = 1, then *D* = 1, no matter what values are taken by the variables in $\vec{W}=\langle B,T\rangle$, and that this remains so even if we build into the antecedent of the relevant counterfactual the additional information that *S* = 1 and/or *D* = 1 holds (that is, even if an arbitrary subset of the variables in $\vec{Z}$ were to take the original values that they received in the context {CI=1,BI=1}). For instance it is true that

(PE,{CI=1,BI=1})⊨[C=1∧B=1∧T=0∧S=1]D=1.

That is, given the model and the context, *D* would have taken value *D* = 1 if *C* had taken its actual value, *C* = 1, while *B* and *T* had taken the values *B* = 1 and *T* = 0, even if *S* had taken its actual value, *S* = 1.

So AC2(b) is satisfied. We have already seen that AC1, AC3, and AC2(a) are satisfied. Thus AC yields the correct verdict that *C* = 1 (Corleone’s order) is an actual cause of *D* = 1 (McCluskey’s death).

AC also yields the correct verdict that *B* = 1 (Barzini’s order) is not an actual cause of *D* = 1. In order to get the sort of contingent dependence of *D* = 1 upon *B* = 1 required by condition AC2(a), it will be necessary for *S* to take the non-actual value *S* = 0. The trouble is that if it were also the case that certain subsets of the variables on the Barzini process were to take their actual values (in particular, the set {*T*}), then variable *D* would take the value *D* = 0, contrary to the requirement of condition AC2(b).

For example, consider the obvious partition $\vec{Z}=\langle B,T,D\rangle$ and $\vec{W}=\langle C,S\rangle$, and consider the assignment $\vec{w}\prime =\{C=1,S=0\}$. Condition AC2(a) is satisfied for this partition and this assignment.^24^ In particular, it is true that

(PE,{CI=1,BI=1})⊨[B=0∧C=1∧S=0]¬D=1.

That is to say, in this model and context, if Barzini hadn’t issued his order and Corleone had issued his order, but Sonny hadn’t shot, then McCluskey wouldn’t have died.

But notice that AC2(b) is not satisfied for this partition and assignment of values to $\vec{W}$. For take $\vec{Z}\prime =\{T\}\subset \vec{Z}$, and observe that

(PE,{CI=1,BI=1})⊭[B=1∧C=1∧S=0∧T=0]D=1.

That is, in the model and the context, it is false that if *B* had taken its actual value *B* = 1, and the variables in $\vec{W}=\langle C,S\rangle$ had taken the values *C* = 1 and *S* = 0 (the values that they receive under the assignment $\vec{w}\prime$), while the subset $\vec{Z}\prime =\{T\}$ of the variables in $\vec{Z}=\langle B,T,D\rangle$ had taken their actual values—namely, *T* = 0—then it would have been that *D* = 1. Intuitively, it is not the case that if Barzini had issued his order, Corleone had issue his order, but Sonny hadn’t shot, and (as was actually the case) Turk hadn’t shot, then McCluskey would have died.

Nor is there any other partition $\left(\vec{Z},\vec{W}\right)$ of the endogenous variables {C,B,S,T,D} such that AC2 is satisfied. In particular, none of the remaining variables on the Barzini process, {*T*, *D*}, can be assigned to $\vec{W}$ instead of $\vec{Z}$, for the values of each of these variables ‘screens off’ *B* from *D*. The result would be that for any assignment $\vec{w}$ of values to the variables in $\vec{W}$, not both AC2(a) and AC2(b) are satisfied. On the other hand, at least one of the variables on the initial Corleone process {*C*, *S*}, must be an element of $\vec{W}$, since only by supposing that such a variable takes a value of zero do we get the contingent dependence required by AC2(a). But reassigning the other variable to $\vec{Z}$ will not affect the fact that AC2(b) fails to hold: it will remain true that if *B* = 1 but *T* = 0, and some variable on the Corleone process had taken a value of zero, then it would have been that *D* = 0, so that AC2(b) is violated.

So AC gives the correct diagnosis of this sort of preemption. It does so, intuitively, on the correct grounds. Specifically, the reason Corleone’s order is counted as a cause is that (i) given Turk’s non-shooting, McCluskey’s death depends upon Corleone’s order; and (ii) there is a complete causal process running from Corleone’s order to McCluskey’s death, as indicated by the fact that for arbitrary subsets of events on the Corleone process, it is true that if Corleone had issued his order, and Turk hadn’t shot, and those events had occurred, then McCluskey would have died.

By contrast, Barzini’s order isn’t counted as a cause because although (i) given Sonny’s non-shooting, McCluskey’s death counterfactually depends upon Barzini’s order,^25^ nevertheless, (ii) there is no complete causal process from Barzini’s order to McCluskey’s death as indicated by the fact that, for example, if Barzini issued his order and Sonny didn’t shoot but (as was actually the case) Turk didn’t shoot, then McCluskey would have survived.

The example that we have been considering is one of so-called early preemption. Halpern and Pearl show how their account provides similarly intuitive treatments of symmetric overdetermination and partial causation (Halpern and Pearl [2001], pp. 197–8, [2005], pp. 856–8), hastening and delaying (Halpern and Pearl [2001], pp. 198–9, [2005], pp. 859–60), late preemption (Halpern and Pearl [2001], pp. 199–200, [2005], pp. 861–4), causation by omission (Halpern and Pearl [2005], pp. 865–7), double prevention (Halpern and Pearl [2005], pp. 867–9), and a range of other cases (Halpern and Pearl [2001], pp. 200–2).

There are, however, some more subtle cases that they claim their definition does not diagnose correctly (Halpern and Pearl [2005], pp. 869–77). They take the view that, as it stands, AC is too liberal. They attempt to deal with the problem cases (Halpern and Pearl [2005], p. 870) by appealing to the notion of an extended causal model. This is simply defined as an ordered pair, 〈〈V,E〉,A〉, where 〈V,E〉 is an SEM, and A is a set of ‘allowable’ settings for the endogenous variables, Y⊂V.^26^ A setting of a subset of the endogenous variables is allowable if it can be extended to a setting in A. The idea, then, is to require that the variable setting $\vec{W}=\vec{w}\prime$, appealed to in condition AC2 of their definition AC, be an allowable setting. Halpern and Pearl wish to count as non-allowable those settings that correspond to ‘unreasonable’ (Halpern and Pearl [2005], p. 869) or ‘fanciful’ (Halpern and Pearl [2005], p. 870) scenarios.

Elsewhere in the structural equations literature, attempts have been made to analyse actual causation in terms of SEMs that represent only ‘serious possibilities’ (Hitchcock [2001a], pp. 287, 294, 298; Woodward [2005], pp. 86–91). More recently, attempts have been made to provide a more rigorous account of allowable settings in terms of normality rankings over possible worlds (Halpern [2008], pp. 203–5; Halpern and Hitchcock [2010], pp. 400–3, [2015], Section 6; cf. Halpern and Pearl [2001], p. 202).

We needn’t go into the details here. The cases that are claimed to require a restriction to allowable settings tend to be rather subtle. Perhaps a fully adequate analysis of probabilistic actual causation would require a similar restriction. It seems plausible that the criteria for allowable settings that have been developed in the literature on deterministic actual causation carry over to the probabilistic case. Indeed, one of the criteria for normality that has been suggested is statistical frequency (Halpern and Hitchcock [2010], p. 402, [2015], pp. 429–30); clearly such a notion is applicable in a probabilistic context. Yet, Halpern ([unpublished], Section 1) and Blanchard and Schaffer ([forthcoming], Section 3) have raised doubts about the need to supplement Halpern and Pearl’s account with a normality-based restriction on allowable settings. Consequently, I will just focus upon extending the unrestricted version of their definition to the probabilistic case here.

A modification of AC that I will consider in some detail (because it is very plausible, and plausibly ought to be carried across to the probabilistic case too) is what Halpern and Pearl ([2005], p. 859) call ‘a contrastive extension to the definition of cause’. It is rather plausible that actual causation is contrastive in nature (Hitchcock [1996a], [1996b]; Schaffer [2005], [2013]). Often, our judgements of actual causation, rather than taking the form ‘$\vec{X}=\vec{x}$ actually caused ϕ’, instead take the form ‘$\vec{X}=\vec{x}$ rather than $\vec{X}=\vec{x}\prime$ actually caused ϕ rather than $\phi^{\prime }$’, where $\vec{x}\neq \vec{x}\prime$ and ϕ is incompatible with $\phi^{\prime }$ (Halpern and Pearl [2005], p. 859). Or, more generally, ‘$\vec{X}=\vec{x}$ rather than $\vec{X}=\vec{x}\prime$ actually caused ϕ rather than ϕ′’, where $\vec{X}=\vec{x}\prime$ denotes a set of formulas of the form $\vec{X}=\vec{x}\prime$ such that for each such formula, $\vec{x}\neq \vec{x}\prime$, and where ϕ′ represents a set of formulas of the form $\phi^{\prime }$ such that for each such formula, ϕ is incompatible with ϕ′ (cf. Schaffer [2005], pp. 327–8). Following Schaffer ([2005], p. 329), I will call $\vec{X}=\vec{x}\prime$ and ϕ′ ‘contrast sets’. The view that actual causation is contrastive both on the cause and on the effect side is thus the view that actual causation is a quaternary relation (Schaffer [2005], p. 327, [2013], p. 46) with $\vec{X}=\vec{x},\;\vec{X}=\vec{x}\prime ,\;\phi$, and ϕ′ as its relata, rather than a binary relation with just $\vec{X}=\vec{x}$ and ϕ as its relata.^27^ The suggestion is that claims like ‘$\vec{X}=\vec{x}$ is an actual cause of ϕ’ are incomplete and liable to be ambiguous, since no contrast sets are explicitly specified.^28^

To illustrate the plausibility of the view that actual causation is contrastive, consider a case where Doctor can administer no dose, one dose, or two doses of medicine to Patient. Patient will fail to recover if no dose is administered, but will recover if either one or two doses are administered. Let us suppose that Doctor in fact administers two doses, and Patient recovers. It would be natural to model this causal scenario using a ternary variable *M*, which takes value 0, 1, or 2 according to whether Doctor administers 0, 1, or 2 doses of medicine, and a binary variable, *R*, which takes value 0 if Patient fails to recover and 1 if she recovers. We can also add an exogenous variable, *I*, which takes value 0 if Doctor intends to administer zero doses, 1 if Doctor intends to administer one dose, and 2 if Doctor intends to administer two doses. The three structural equations for this case are then *I* = 2, *M* = *I*, and R=M/Max{M,1}. The actual solution is *I* = 2, *M* = 2, and *R* = 1.

I think that the natural reaction to the claim, ‘Doctor’s administering two doses of Medicine caused Patient to recover’, is one of ambivalence (at least if there are no further contextual factors to pick out one of the two alternative actions available to Doctor as the relevant one). While one of the alternative actions available to Doctor (*M* = 0) would have made a difference to whether or not Patient recovered, the other (*M* = 1) would have made no difference. A natural interpretation of our ambivalent attitude is that causation is contrastive in nature, and that ‘Doctor’s administering two doses of Medicine caused Patient to recover’ is ambiguous between ‘Doctor’s administering two doses rather than no doses of Medicine caused Patient to recover’ (to which most people would presumably assent) and ‘Doctor’s administering two doses rather than one dose of Medicine caused Patient to recover’ (to which most people would presumably not assent).

Yet, as it stands, AC unequivocally yields the result that *M* = 2 was an actual cause of *R* = 1. Suppose that $\vec{X}=\{M\},\;\vec{x}=\{M=2\},\;\vec{x}\prime =\{M=0\}$, and that ϕ is *R* = 1. Since *M* = 2 and *R* = 1 are the values of *M* and *R* in the solution to the structural equations of the model described, given the actual context AC1 is satisfied. Since $\vec{X}=\{M\}$ has no (non-empty) strict subsets, AC3 is satisfied. To see that AC2 is satisfied, consider the partition ($\vec{Z},\;\vec{W}$) of the endogenous variables in our model such that $\vec{Z}=\langle M,R\rangle$ and $\vec{W}=Ø$. Condition AC2(a) will be satisfied if, for some assignment of values to the variables in $\vec{W}$, it is true that if the variables in $\vec{W}$ had taken those values and *M* had taken value *M* = 0, then *R* would have taken value *R* = 0. Since there are no variables in $\vec{W}$, AC2(a) reduces to the requirement that if *M* had taken value *M* = 0, then *R* would have taken the value *R* = 0. Since our model implies that this is so, AC2(a) is satisfied. Finally, condition AC2(b) is rather trivially satisfied. Since there are no variables in $\vec{W}$ or in $\vec{Z}\setminus M,R$, AC2(b) just reduces to the requirement that if it had been that *M* = 2, then it would have been that *R* = 1. Since our model implies that this is so, AC2(b) is satisfied. Since, as we have seen, AC1, AC2(a), and AC3 are also all satisfied, AC yields the result that *M* = 2 is an actual cause of *R* = 1.

AC is unequivocal that *M* = 2 is a cause of *R* = 1, whereas intuition is equivocal. It would thus seem desirable to modify AC to bring it into closer alignment with intuition. Specifically, it would seem desirable to adjust AC so that it can capture the nuances of our contrastive causal judgements (Halpern and Pearl [2005], p. 859). This is easily achieved. To turn AC into an analysis of $\vec{X}=\vec{x}$ rather than $\vec{X}=\vec{x}\prime$ being an actual cause of ϕ, we simply need to require that AC2(a) hold not just for some non-actual setting of $\vec{X}$, but for precisely the setting $\vec{X}=\vec{x}\prime$ (cf. Halpern and Pearl [2005], p. 859). More generally, to turn AC into an analysis of $\vec{X}=\vec{x}$ rather than $\vec{X}=\vec{x}\prime$ being an actual cause of ϕ, where $\vec{X}=\vec{x}\prime$ denotes a set of formulas of the form $\vec{X}=\vec{x}\prime$, we simply need to require that AC2(a) hold for every formula of the form $\vec{X}=\vec{x}\prime$ in $\vec{X}=\vec{x}\prime$.

This gives the correct results in the example just considered. The reason that the original version of AC yielded the unequivocal result that *M* = 2 is an actual cause of *R* = 1 is that the original version of AC2(a) requires simply that there be some other alternative value of *M* such that if *M* had taken that alternative value (and the variables in $\vec{W}$ had taken some possible assignment), then it would have been the case that *R* = 0. This condition is satisfied because *M* = 0 is such a value. The revised version of AC just proposed does not give an unequivocal result about whether *M* = 2 is an actual cause of *R* = 1. Indeed, it doesn’t yield any result until a contrast set for *M* = 2 is specified.

The revised version of AC does yield the verdict that *M* = 2 rather than *M* = 0 was an actual cause of *R* = 1. Specifically, taking the contrast set to be {M=0}, the revised version of AC is satisfied for precisely the same reason that taking $\vec{X}=\vec{x}\prime$ to be *M* = 0 allowed us to show that the original version of AC is satisfied when we consider *M* = 2 as a putative cause of *R* = 1. The revised version of AC also yields the verdict that *M* = 2 rather than *M* = 1 is not a cause of *R* = 1. This is because the revised version of AC2(a) is violated when we take {M=1} to be the contrast set. Specifically, it’s not the case that if *M* had taken the value *M* = 1 (and the variables in $\vec{W}$ had taken some possible assignment—a trivially satisfied condition in this case because $\vec{W}=Ø$),^29^ then variable *R* would have taken *R* = 0. The revised AC thus gives the intuitively correct results about these contrastive causal claims. Moreover, it can explain the equivocality of intuition about the claim ‘*M* = 2 was an actual cause of *R* = 1’ in terms of its ambiguity between ‘*M* = 2 rather than *M* = 0 was an actual cause of *R* = 1’ (which it evaluates as true) and ‘*M* = 2 rather than *M* = 1 was an actual cause of *R* = 1’ (which it evaluates as false).

As suggested above, we may find it plausible to build contrast in on the effect side too (Schaffer [2005], p. 328; Woodward [2005], p. 146). To change our previous example somewhat, suppose that one dose of medicine leads to speedy recovery, two doses leads to slow recovery (two doses is an ‘overdose’ that would adversely affect Patient’s natural immune response), while zero doses leads to no recovery. Suppose that Doctor in fact administers two doses, and so Patient recovers slowly. In this case, we might reasonably represent the outcome using a variable that has three possible values: *R* = 0 represents no recovery, *R* = 1 represents speedy recovery, and *R* = 2 represents slow recovery. Taking *M* and *I* to be variables with the same possible values (with the same interpretations) as before, the structural equations for this new case are *I* = 2, *M* = *I*, and *R* = *M*. The actual solution is *I* = 2, *M* = 2, and *R* = 2.

We might wish to have the capacity to analyse causal claims like ‘Doctor’s administering two doses rather than one dose of Medicine caused Patient to recover slowly rather than quickly’. It is unproblematic to modify AC to achieve this. In order to analyse a claim of the form ‘$\vec{X}=\vec{x}$ rather than $\vec{X}=\vec{x}\prime$ actually caused ϕ rather than ϕ′’ we simply need to replace ¬ϕ with ϕ′ in condition AC2(a) (Halpern and Pearl [2005], p. 859) and require that the modified AC2(a) hold not just for some non-actual setting of $\vec{X}$, but for precisely the setting $\vec{X}=\vec{x}\prime$ (cf. Halpern and Pearl [2005], p. 859). This yields the correct result in the present case because while the actual value of *M* is *M* = 2 and the actual value of *R* is *R* = 2, it is true that if *M* had taken the value *M* = 1, then *R* would have taken the value *R* = 1.

More generally, suppose that we wish to analyse claims of the form ‘$\vec{X}=\vec{x}$ rather than $\vec{X}=\vec{x}\prime$ actually caused ϕ rather than ϕ′’, where $\vec{X}=\vec{x}\prime$ denotes a set of formulas of the form $\vec{X}=\vec{x}\prime$ such that for each such formula, $\vec{x}\neq \vec{x}\prime$, and where ϕ′ represents a set of formulas of the form ϕ′ such that for each such formula, ϕ is incompatible with ϕ′. To do this, we simply need to require that for each formula of the form $\vec{X}=\vec{x}\prime$ in $\vec{X}=\vec{x}\prime$, there is some formula of the form ϕ′ in ϕ′ such that AC2(a) holds when ¬ϕ is replaced with ϕ′, and the non-actual setting of $\vec{X}$ appealed to in AC2(a) is taken to be precisely the setting $\vec{X}=\vec{x}\prime$ (cf. Schaffer [2005], p. 348).^30^

This revised definition reduces to the original AC in the case where the putative cause is primitive event *X* = *x* (rather than a conjunction of primitive events), and the putative effect is primitive event *Y* = *y* (rather than an arbitrary Boolean combination of primitive events), and the variables *X* and *Y* representing those primitive events are binary, with their alternative possible values being X=x′ and Y=y′ (x≠x′, y≠y′). In such a case, the setting $\vec{X}=\vec{x}$ of the putative cause variables appealed to in the unmodified AC is just the setting *X* = *x*, and the variable ϕ representing the putative effect is simply to be replaced by *Y* = *y*. Since, in this case, there is only one possible but non-actual value of *X*—namely, the value x—′X=x′ is automatically the non-actual setting of the putative cause variable appealed to in the unmodified AC2(a). Likewise, in such a case, ¬ϕ (which appears in AC2(a)) just means ¬Y=y, which, because *Y* is binary, just corresponds to Y=y′. Moreover, in such a case, {X=x′} and {Y=y′} automatically serve as the contrast sets appealed to in AC2(a) where AC is modified (in the way suggested in the previous paragraph) to incorporate contrastivity. This is because there are no other possible but non-actual values of the putative cause and effect variables. So, under these circumstances, both the original and revised version of AC2(a) require the same thing, namely, that *Y* would take the value Y=y′ if *X* were to take the value X=x′ and the variables $\vec{W}$ were to take the values $\vec{W}=\vec{w}\prime$. Since the modified and unmodified versions of AC differ only in AC2(a), it follows that both versions of the analysis will yield the same results in such cases.

This explains why the unmodified definition AC works well in our preemption scenario, where binary variable *C* taking value *C* = 1 (representing Corleone’s order) is considered as a putative cause of binary variable *D* taking value *D* = 1 (representing McCluskey’s death). Since, where the cause and effect variables are binary, the relevant contrasts are selected automatically, saying that *C* = 1 is an actual cause of *D* = 1 is effectively equivalent to saying that *C* = 1 rather than *C* = 0 is an actual cause of *D* = 1 rather than *D* = 0.

In closing this section, it is worth noting that although the causal notion upon which Halpern and Pearl ([2001], [2005]) focus is that of actual causation, other causal notions can be fruitfully analysed in the SEM framework. In fact, Pearl ([2009]), Hitchcock ([2001b]), and Woodward ([2005]) analyse a range of causal notions in terms of SEMs, including ‘net effect’ (Hitchcock [2001b], p. 372), ‘total cause’ (Woodward [2005], p. 51), ‘component effect’ (Hitchcock [2001b], pp. 374, 390–5), ‘direct cause’ (Woodward [2005], p. 55), ‘direct effect’ (Pearl [2009], pp. 126–8), and ‘contributing cause’ (Woodward [2005], p. 59). While my interest in this article is with actual causation rather than these other causal notions, I do think that there is another causal notion that is very closely related to that of actual causation, and which can be defined simply as a corollary to (the modified) AC, namely, that of ‘prevention’. I’m inclined to think that prevention is just the flip-side of actual causation. Specifically, it seems plausible to me that, if (by the lights of the modified AC) $\vec{X}=\vec{x}$ (rather than $\vec{X}=\vec{x}\prime$) is an actual cause of ϕ rather than ϕ′, then $\vec{X}=\vec{x}$ (rather than $\vec{X}=\vec{x}\prime$) prevents ϕ′ rather than ϕ from happening. I shall discuss the issue of probabilistic prevention in Section 8.

## 6 The Probabilistic Case

In attempting to analyse probabilistic actual causation, philosophers have typically appealed to the notion of ‘probability raising’. The idea is that, at least when circumstances are benign—for example, when there are no preempted potential causes of the effect—an actual cause raises the probability of its effect.^31^ Turning this insight into a full-blown analysis of probabilistic actual causation depends, among other things, upon giving an account of what it is for circumstances to be ‘benign’ (ideally, an account that does not itself appeal to actual causation). This is part of what I shall seek to do below, drawing inspiration from Halpern and Pearl’s account of actual causation in the deterministic case.^32^

But first it is worth considering in a bit more detail precisely what the notion of probability raising amounts to. In this context, some notation introduced by Godszmidt and Pearl ([1992], pp. 669–70; see also Pearl [2009], pp. 23, 70, 85) is helpful. In that notation, $do(\vec{V}=\vec{v})$ represents the set of variables, $\vec{V}$ coming to have the values $\vec{V}=\vec{v}$ as a result of ‘local surgeries’ (Pearl [2009], p. 223)—or (just as good) as a result of Woodwardian ‘interventions’ (Woodward [2005], p. 98), or Lewisian ‘small miracles’ (Lewis [1979]), p. 468ff)—as opposed to $\vec{V}$ coming to have the values $\vec{V}=\vec{v}$ as a result of different initial conditions operating via ordinary causal processes.^33^

Suppose that $\vec{X}=\vec{x}$ is a candidate actual cause and ϕ is a putative effect of $\vec{X}=\vec{x}$. One way of cashing out the idea that variables $\vec{X}$ taking the values $\vec{X}=\vec{x}$ rather than $\vec{X}=\vec{x}\prime$ raises the probability of ϕ is in terms of the following inequality:

(1) $$P(\phi |do(\vec{X}=\vec{x}))>P(\phi |do(\vec{X}=\vec{x}\prime )).$$

This says that the probability of ϕ that would obtain if $\vec{X}$ were to be set to $\vec{X}=\vec{x}$ by interventions (or by local surgeries or small miracles)^34^ is higher than the probability of ϕ that would obtain if $\vec{X}$ were to be set to $\vec{X}=\vec{x}\prime$ by interventions.^35^ Note that $P(\phi |do(\vec{X}=\vec{x}))$ thus represents something different from $P(\phi |\vec{X}=\vec{x})$. The latter is an ordinary conditional probability: the probability that ϕ obtains conditional upon $\vec{X}=\vec{x}$ obtaining. The former, by contrast, represents a counterfactual probability: the probability for ϕ that would obtain if the variables $\vec{X}$ had been set to the values $\vec{X}=\vec{x}$ by interventions

The counterfactual probability, $P(\phi |do(\vec{X}=\vec{x}))$, is liable to diverge from the conditional probability, $P(\phi |\vec{X}=\vec{x})$; witness the difference between the probability of a storm conditional upon the barometer needle pointing towards the word ‘storm’, on the one hand, and the probability that there would be a storm if I intervened upon the barometer needle to point it towards the word ‘storm’, on the other (cf. Pearl [2009], pp. 110–11).

One of the advantages of appealing to counterfactual probabilities rather than to conditional probabilities in analysing actual causation is precisely that when the counterfactuals in question are given a suitably non-backtracking semantics (that is, where their antecedents are taken to be realized by interventions, small-miracles, local surgeries, or the like), we avoid generating probability-raising relations between independent effects of a common cause (see Lewis [1986a], p. 178). For example, the probability of a storm is higher conditional upon the barometer needle pointing to the word ‘storm’ than it is conditional upon the barometer needle’s not doing so (cf. Salmon [1984], pp. 43–4). This is not because the barometer reading is an actual cause of the storm, but rather because an earlier fall in atmospheric pressure is very probable conditional upon the needle of the barometer pointing towards ‘storm’, and a storm is very probable conditional upon a fall in atmospheric pressure. By contrast, it is false that the probability of a storm would be higher if I were to intervene to point the barometer needle towards ‘storm’ than if I were to intervene to point it towards some other word (for example, ‘sun’), precisely because my intervention breaks the normal association between the atmospheric pressure and the barometer reading. Understanding probability raising in terms of (non-backtracking) counterfactuals thus ensures the elimination of probability-raising relationships that are due merely to common causes.

Another advantage of appealing to counterfactual probabilities rather than conditional probabilities in analysing actual causation is that we retain the possibility of applying our probabilistic analysis of actual causation to the deterministic case (cf. Lewis [1986a], pp. 178–9). Under determinism, an effect, ϕ, counterfactually depends upon its cause, $\vec{X}=\vec{x}$, when circumstances are benign (that is, where ϕ isn’t overdetermined, and where $\vec{X}=\vec{x}$ doesn’t preempt a potential alternative cause $\vec{Y}=\vec{y}$ of ϕ). In the probabilistic case, ϕ might merely have its probability raised by $\vec{X}=\vec{x}$ in such circumstances. This is because in the probabilistic case, it may well be that ϕ would have had a residual background chance of occurring, even if $\vec{X}=\vec{x}$ had been absent. For example, the probability that an atom will decay within a given interval of time can in some cases be increased by bombarding it with neutrons. If the atom decays within the relevant time interval, then we might reasonably say that the bombardment was an actual cause. Still, if the bombarded atom was already unstable, it is not true that if it hadn’t been bombarded, then it wouldn’t have decayed within the relevant time interval: it still might have decayed (there would have been a positive—and perhaps even reasonably high—chance of its doing so), it’s just that the probability of its doing so would have been lower than it actually was (cf. Lewis [1986a], p. 176).

Still, if probability raising is understood in terms of inequalities like Inequality (1), then counterfactual dependence can be seen as a limiting case of probability raising. Specifically, suppose that ϕ and $\vec{X}=\vec{x}$ actually obtain and that it is true that if, due to an intervention, $\vec{X}=\vec{x}\prime$ (rather than $\vec{X}=\vec{x}$) had obtained, then ¬ϕ would have obtained. Plausibly, it follows that $P(\phi |do(\vec{X}=\vec{x}\prime ))=0$—that is, that if $\vec{X}=\vec{x}\prime$ had obtained (due to an intervention), then the chance of ϕ would have been zero. After all, if the chance of ϕ would have been greater than zero, then it is not true that ¬ϕ would have obtained (Lewis [1986a], p. 176).^36^

Counterfactual dependence of ϕ upon $\vec{X}=\vec{x}$ also requires that if $\vec{X}=\vec{x}$ had obtained, then ϕ would have obtained. That is, it requires that $\vec{X}=\vec{x}□\to \phi$ (or $[\vec{X}=\vec{x}]\phi$ in the notation adopted here). But it very plausibly follows from $[\vec{X}=\vec{x}]\phi$ that $P(\phi |do(\vec{X}=\vec{x}))>0$. Denying this would require accepting that it could be the case that if $\vec{X}=\vec{x}$ had occurred, then ϕ would have occurred, even though the probability of ϕ occurring would have been equal to zero.^37^ Putting these two results together, we get that where ϕ and $\vec{X}=\vec{x}$ occur (which is a necessary condition for their standing in an actual causal relation), if ϕ counterfactually depends upon its being the case that $\vec{X}=\vec{x}$ rather than $\vec{X}=\vec{x}\prime$, then Inequality (1) holds. Counterfactual dependence is thus a special case of the sort of probabilistic dependence captured by Inequality (1).

As hinted at above, we can think of analyses of deterministic actual causation in terms of SEMs, such as Halpern and Pearl’s, as starting with the insight that effects counterfactually depend upon their actual causes when circumstances are benign, and then giving an account of what variables must be held fixed at which values in order to recover benign circumstances (and therefore contingent counterfactual dependence) even where actual circumstances are unbenign. The probabilistic analysis of actual causation developed below starts with the idea that effects have their probability raised by their actual causes when circumstances are benign, and then gives an account of what variables must be held fixed at which values in order to recover benign circumstances (and therefore contingent probability raising) even where actual circumstances are unbenign.^38^ Given the structural analogy between the two sorts of account, with probability raising playing the role in the one account that counterfactual dependence plays in the other, if counterfactual dependence is a limiting case of probability raising, then the prospects of a unified treatment of deterministic and probabilistic actual causation look good.

If we cashed out the notion of probability raising, not in terms of the counterfactual probabilities that appear in Inequality (1), but rather in terms of an inequality between conditional probabilities—$P(\phi |\vec{X}=\vec{x})>P(\phi |\vec{X}=\vec{x}\prime )$—then it would be much less clear that deterministic causation could be treated as a limiting case of probabilistic causation (cf. Lewis [1986a], pp. 178–9). The trouble is that under determinism, it is plausible that causes may have a chance of one of occurring (given initial conditions). Indeed, the putative causes in the deterministic preemption scenario described in Section 2 (namely, Corleone’s order and Barzini’s order) were taken to follow deterministically from the context (and thus to have a chance of one given that context). But where $P(\vec{X}=\vec{x})=1$, then where $\vec{x}\neq \vec{x}\prime ,\;P(\vec{X}=\vec{x}\prime )=0$ and—according to standard probability theory—$P(\phi |\vec{X}=\vec{x}\prime )$ is undefined. So our probabilistic analysis of actual causation will run into trouble in the deterministic case if we understand the notion of probability raising in terms of the inequality $P(\phi |\vec{X}=\vec{x})>P(\phi |\vec{X}=\vec{x}\prime )$. There is no such problem if we understand probability raising in terms of Inequality (1), since the fact that $P(\vec{X}=\vec{x})=1$ does not imply that the counterfactual probability $P(\phi |do(\vec{X}=\vec{x}\prime ))$ (where $\vec{x}\neq \vec{x}\prime$) is undefined.

It is worth emphasizing that, not only is $P(\phi |do(\vec{X}=\vec{x}\prime ))$ not the same as $P(\phi |\vec{X}=\vec{x}\prime )$, the former isn’t a conditional probability at all. $P(\cdot |do(\vec{X}=\vec{x}\prime ))$ is simply a different probability distribution than P(·); we could just as well denote these distributions ‘$P_{1}(\cdot )$’ and ‘$P_{2}(\cdot )$’. In particular, $P(\phi |do(\vec{X}=\vec{x}\prime ))$ isn’t defined in terms of P(·) via the ratio definition of conditional probability, that is, it is not the case that $P(\phi |do(\vec{X}=\vec{x}\prime ))=P(\phi \text{\&}do(\vec{X}=\vec{x}\prime ))/P(do(\vec{X}=\vec{x}\prime ))$. This could not be the case, since $do(\vec{X}=\vec{x}\prime )$ (unlike $\vec{X}=\vec{x}\prime$) is not an event in the probability space over which P(·) is defined (see Pearl [1995], pp. 684–5, [2009], pp. 109–11, 332, 386, 421–2, Woodward [2005], pp. 47–8). Rather, P(·) is the actually obtaining probability distribution on the field of events generated by our variable set V (of which the variables in $\vec{X}$ and those in ϕ are subsets), whereas $P(\cdot |do(\vec{X}=\vec{x}\prime ))$ is the probability distribution (on that same field of events) that would obtain if the variables in $\vec{X}$ were set to the values $\vec{X}=\vec{x}\prime$ by interventions. Thus, Pearl ([2009], p. 110) suggests that we can construe the intervention do(·) as a function that takes the actual probability distribution P(·) and a possible event $\vec{X}=\vec{x}\prime$ as an input and yields the counterfactual probability distribution $P(\cdot |do(\vec{X}=\vec{x}\prime ))$ as an output.

I have suggested that, when circumstances are benign, actual causation might involve probability raising. Yet, actual causation cannot simply be identified with the probability raising of one event by another. This is because circumstances aren’t always benign. Preemption cases are among the cases in which circumstances aren’t benign. It was seen in Section 2 that deterministic preemption cases show that counterfactual dependence (even under determinism) is not necessary for actual causation. Probabilistic preemption cases show that probability raising is not necessary for actual causation either. Interestingly, such cases also show that probability raising is not sufficient for actual causation (Menzies [1989], pp. 645–7; Menzies [1996], pp. 88–9). This is in contrast to the deterministic case, where counterfactual dependence, arguably, is sufficient for actual causation. We can describe a probabilistic preemption case by simply modifying our earlier deterministic preemption scenario. The modified scenario is as follows:

PE${}^{*}$: The New York Police Department is due to go on parade at the parade ground on Saturday. Knowing this, Don Corleone decides that, when Saturday comes around, he will order Sonny to go to the parade ground and shoot and kill Police Chief McCluskey. Not knowing Corleone’s plan, Don Barzini decides that when Saturday comes around, he will order Turk to shoot and kill McCluskey. To simplify, suppose that each of the following chances is negligible: the chance of each of the dons not issuing his order given his intention to do so, the chance of Turk or Sonny shooting McCluskey if not ordered to do so, the chance of McCluskey dying unless he is hit by either Turk’s or Sonny’s bullet, and the chance of Turk shooting if Sonny shoots. Suppose that Sonny is a fairly obedient type, and that his opportunity to shoot will (with a chance approximating one) come earlier than Turk’s (since Corleone’s headquarters are closer to the police parade ground than Barzini’s headquarters). Let us assume that, given Corleone’s order, there is a 0.9 chance that Sonny will shoot McCluskey. Sonny, however, is not a great shot and if he shoots, there’s only a 0.5 chance that he’ll hit and kill McCluskey. Turk is also obedient, but will (with a chance approximating one) have the opportunity to shoot only if Sonny doesn’t shoot (even if Sonny shoots and misses, McCluskey will almost certainly be whisked away to safety before Turk gets a chance to shoot). But if Barzini issues his order and Sonny does not shoot, then there is a 0.9 chance that Turk will shoot. And if Turk shoots, there is a 0.9 chance that he will hit and kill McCluskey. Suppose that, in actual fact, both Corleone and Barzini issue their orders. Sonny arrives at the parade ground first, shooting and killing McCluskey. Turk arrives on the scene afterwards and doesn’t shoot.

Intuitively, just as in the deterministic scenario, Corleone’s order was a cause of McCluskey’s death, while Barzini’s order was not a cause. Still, the chance of McCluskey’s death if Corleone issued his order was:

(2) P(D=1|do(C=1))≈(0.9×0.5)+(0.1×(0.9×0.9))=(0.45)+(0.081)=0.531.

That is (given the stipulations of the example), the chance of McCluskey’s death if Corleone issues his order is approximately equal to the probability that Sonny shoots if Corleone issues his order (0.9), multiplied by the probability that Sonny hits and kills McCluskey if he shoots (0.5), plus the probability that Sonny doesn’t shoot if Corleone issues his order (0.1), multiplied by the product of the probability that Turk shoots if Sonny doesn’t (0.9), and the probability that Turk hits and kills McCluskey if he shoots (0.9).

By contrast, the chance of McCluskey’s death if Corleone had not issued his order would have been:

(3) P(D=1|do(C=0))≈0.9×0.9=0.81.

That is (given the stipulations of the example), the chance of McCluskey’s death if Corleone had not issued his order would be approximately equal to the chance that Turk would shoot if Barzini issued his order and Sonny had not shot (0.9), multiplied by the probability that Turk would hit and kill McCluskey if he shot (0.9).

It is worth noting that in evaluating these probabilities, there is no need to explicitly hold fixed the context—namely, the intentions of the dons to issue their orders, CI=1&BI=1—by including it as an argument in the do(.) function in the counterfactual probability expressions that appear on the left-hand side of the approximate equalities (Approximate Equalities (2) and (3)) (so that the expression on the left-hand side of Approximate Equality (3), for example, becomes P(D=1|do(C=0&CI=1&BI=1))). This is because the context is already held fixed, implicitly, in virtue of the non-backtracking nature of the counterfactuals. In evaluating the counterfactual probability expressed by Approximate Equality (3), for example, we are to consider a world in which *C* is set to *C* = 0 by an intervention (or local surgery or small miracle) that leaves the context, CI=1&BI=1, undisturbed. The same point applies to all of the counterfactual probabilities considered below.

It follows immediately from Approximate Equalities (2) and (3) that in spite of our intuitive judgement that Corleone’s order was a cause of McCluskey’s death, the former actually lowers the probability of the latter. Specifically,

(4) P(D=1|do(C=1))≈0.531<0.81≈P(D=1|do(C=0)).

Intuitively, the reason why Corleone’s order lowers the probability of McCluskey’s death is that Turk is by far the more competent assassin, and a botched assassination attempt by the relatively incompetent Sonny would prevent Turk from getting an opportunity to attempt the assassination. So although Corleone’s order was an actual cause of McCluskey’s death (because Sonny succeeded), Corleone’s order lowered the probability of McCluskey’s death (because it raised the probability that Sonny would carry out a botched attempt that would prevent the far more competent Turk from taking a shot). The example thus illustrates the well-known fact that causes need not raise the probability of their effects.^39^

Also well-known is the fact that an event can have its probability raised by another event that is not among its causes.^40^ The above example illustrates this phenomenon too. Since Corleone and Barzini issue their orders independently and since (in the context in which they both form the intention to do so) each does so with a probability of approximately one, the probability of McCluskey’s death if Barzini issues his order is approximately equal to the probability of McCluskey’s death if Corleone issues his order. That is,

(5) P(D=1|do(B=1))≈P(D=1|do(C=1))≈0.531.

However, the probability of McCluskey’s death if Barzini had not issued his order is approximately equal to the probability that Sonny shoots if Corleone orders him to (0.9), multiplied by the probability that Sonny hits and kills McCluskey if he shoots (0.5). That is,

(6) P(D=1|do(B=0))≈0.9×0.5=0.45.

It follows immediately from Approximate Equalities (5) and (6) that Barzini’s order raises the probability of McCluskey’s death. Specifically:

(7) P(D=1|do(B=1))≈0.531>0.45≈P(D=1|do(B=0)).

Intuitively, the reason that Barzini’s order raises the probability of McCluskey’s death is that there is some chance that Sonny will fail to shoot and in such circumstances, given Barzini’s order, there is a (fairly high) chance that Turk will shoot and kill McCluskey instead. Since Barzini’s order is nevertheless not an actual cause of McCluskey’s death, the example thus illustrates the fact that probability raising isn’t sufficient for actual causation (even when we understand probability raising in terms of non-backtracking counterfactuals so as to eliminate the influence of common causes),^41^ as well as not being necessary.

That probability raising is neither necessary nor sufficient for actual causation creates a difficulty for existing attempts to analyse probabilistic actual causation (see Hitchcock [2004]). And, as it stands, Halpern and Pearl’s definition AC does not give the correct diagnosis of probabilistic preemption cases like the one just described. In particular, it fails to diagnose Corleone’s order as a cause of McCluskey’s death. The reason is that it is no longer true (as it was in the deterministic preemption case considered above) that as condition AC2(a) requires, (i) given Turk’s non-shooting (and Barzini’s issuing his order), McCluskey’s death counterfactually depends upon Corleone’s order. After all, if Corleone hadn’t issued his order and (Barzini had issued his order but) Turk hadn’t shot, then McCluskey might still have died (from a heart attack, say). I said that the chance of his dying in these circumstances was negligible, not that it was zero.^42^ Moreover, I made the assumption of a negligible probability of his dying in such a situation only for calculational simplicity. In a probabilistic context, one can revise the example so that the probability is rather large, while still ensuring that Corleone’s order is a non-probability-raising cause and Barzini’s order is a probability-raising non-cause of McCluskey’s death.^43^

Moreover, since the Corleone process is now only probabilistic, it is not true, as condition AC2(b) requires, that (ii) if Corleone had issued his order, and (Barzini had issued his order but) Turk hadn’t shot, then McCluskey would have died. After all, in the probabilistic case, there is some chance that Sonny doesn’t shoot even if Corleone issues his order; it was a stipulation of the example that the chance of Sonny shooting if Corleone issues his order is only 0.9. There is also some chance that Sonny fails to kill McCluskey even if he does shoot; it was a stipulation of the example that the chance of McCluskey dying if Sonny shoots is only around 0.5. So it is not true that if Corleone had issued his order and Turk hadn’t shot, then McCluskey would have died. The chance of his dying under such circumstances is only (approximately) 0.9×0.5=0.45.^44^ So, in general, Halpern and Pearl’s definition AC does not (and is not intended to) deliver the correct results in the probabilistic case.

Nevertheless, it seems *prima facie* plausible that Halpern and Pearl’s account might be extended to provide a satisfactory treatment of probabilistic actual causation by substituting its appeals to contingent counterfactual dependence with appeals to contingent probability raising. Specifically, one might maintain that Corleone’s order was a cause of McCluskey’s death because (i) given Turk’s non-shooting (and Barzini’s order), Corleone’s order raised the probability of McCluskey’s death. After all, given Turk’s non-shooting, the probability of McCluskey’s death would have been lower (approximately zero) if Corleone hadn’t issued his order than if he had (approximately 0.45). Moreover, (ii) there is a complete probabilistic causal process running from Corleone’s order to McCluskey’s death, as indicated by the fact that for arbitrary subsets of events on the Corleone process, it is true that given that Turk didn’t shoot (and Barzini issued his order), if Corleone had shot, and those events had occurred, then the probability of McCluskey’s death would have remained higher than it would have been if Corleone hadn’t issued his order.

By contrast, plausibly Barzini’s order isn’t an actual cause because while it is true that (i) given Sonny’s not shooting (and Corleone’s issuing his order), Barzini’s order raised the probability of McCluskey’s death—specifically, given Sonny’s non-shooting, the probability of McCluskey’s death would have been lower (approximately zero) if Barzini hadn’t issued his order than if he had (approximately 0.81)—it is nevertheless not true that (ii) there is a complete probabilistic causal process from Barzini’s order to McCluskey’s death, as indicated by the fact that, for example, if Sonny hadn’t shot (and Corleone had issued his order) and (as was actually the case) Barzini issued his order but Turk didn’t shoot, then the probability of McCluskey’s death would have been no higher than if Barzini hadn’t issued his order in the first place.

In order to render this suggestion more precise, it will be necessary to appeal to the notion of a ‘probabilistic causal model’.

## 7 Probabilistic Causal Models

As noted in the previous section, Pearl ([2009], p. 110) suggests that we can construe do(·) as a function that takes a probability distribution and a formula of the form $\vec{X}=\vec{x}\prime$ as an input, and yields a new probability distribution, $P(\cdot |do(\vec{X}=\vec{x}\prime ))$, as an output. Thinking of do(·) in these terms, we can construe a probabilistic causal model, $M^{*}$, as an ordered triple 〈V,P,do(·)〉, where V is a set of variables, *P* is a probability distribution defined on the field of events generated by the variables in V, and do(·) is a function that when *P* and any formula the form $\vec{V}\prime =\vec{v}\prime$ for $\vec{V}\prime \subseteq V$ are taken as its inputs, yields as an output a new distribution $P(\cdot |do(\vec{V}\prime =\vec{v}\prime ))$—the probability distribution that would result from intervening upon the variables $\vec{V}\prime$ to set their values equal to $\vec{V}\prime =\vec{v}\prime$.

The variable set V can be partitioned into a set of exogenous variables, U, and a set of endogenous variables, Y. In the probabilistic context, exogenous variable U∈U is such that for no possible value *u* of *U* is there a pair of possible value assignments, $\left\{\vec{T}=\vec{t}\prime ,\vec{T}=\vec{t}\prime \prime \right\}$, to the variables in $\vec{T}=V\setminus U$ such that $P(U=u|do(\vec{T}=\vec{t}\prime ))\neq P(U=u|do(\vec{T}=\vec{t}\prime \prime ))$. That is, *U* is exogenous if and only if the probability of none of the possible values of *U* is affected by interventions on the values of the other variables in V. The endogenous variables, V, are the variables that are not exogenous. An assignment of values $\vec{u}$ to variables $\vec{U}$ in set U of exogenous variables, denoted $\vec{U}=\vec{u}$, is (once again) called a ‘context’.

It was observed in Section 3 that Hitchcock ([2001a], p. 287) takes it to be a condition for the ‘appropriateness’ of an SEM, M, that it ‘entail no false counterfactuals’, by which he means that evaluating counterfactuals with respect to M by means of the equation replacement method doesn’t lead to evaluations of counterfactuals as true when they are in fact false (Hitchcock [2001a], p. 283). We can make an analogous requirement of probabilistic causal models. Specifically, where $M^{*}=\langle V,P,do(\cdot )\rangle$ is our probabilistic causal model, it should be required that for any formula the form $\vec{V}\prime =\vec{v}\prime$, such that $\vec{V}\prime \subseteq V$, the distribution $P(\cdot |do(\vec{V}\prime =\vec{v}\prime ))$ that the function do(·) yields as an output (when *P* and $\vec{V}\prime =\vec{v}\prime$ are its inputs) should be the true objective chance distribution on the field of events generated by the variables in V that would result from intervening upon the variables $\vec{V}\prime$ to set their values equal to $\vec{V}\prime =\vec{v}\prime$.^45^

In modelling our probabilistic preemption scenario, we can take the variable set to comprise the variables *CI*, *BI*, *C*, *B*, *S*, *T*, and *D*, where these variables all have the same possible values (with the same interpretations) as they did in the deterministic case. To be appropriate, our probabilistic causal model 〈{CI,BI,C,B,S,T,D},P,do(·)〉 should satisfy the requirement described in the previous paragraph: where V={CI,BI,C,B,S,T,D}, for any formula of the form $\vec{V}\prime =\vec{v}\prime$ such that $\vec{V}\prime \subseteq V$, the distribution $P(\cdot |do(\vec{V}\prime =\vec{v}\prime ))$ that the function do(·) yields as an output (when *P* and $\vec{V}\prime =\vec{v}\prime$ are its inputs) should be the true objective chance distribution on the field of events generated by the variables in V that would result from intervening upon the variables $\vec{V}\prime$ to set their values equal to $\vec{V}\prime =\vec{v}\prime$. Since I am assuming the probabilities described in the probabilistic preemption example (as outlined in the previous section) to be objective chances, these probabilities should be among those that result from the appropriate inputs to do(·).

We can construct a graphical representation of a probabilistic model, 〈V,P,do(·)〉 by taking the variables in V as the nodes or vertices of the graph and drawing a directed edge (‘arrow’) from a variable *V_i_* to a variable *V_j_* ($V_{i},V_{j}\in V$) just in case, where $\vec{S}=V\setminus V_{i},V_{j}$, there is some assignment of values $\vec{S}=\vec{s}\prime$, some pair of possible values $\left\{v_{i},v_{i}\prime \right\}$ ($v_{i}\neq v_{i}\prime$) of *V_i_*, and some possible value *v_j_* of *V_j_* such that $P(V_{j}=v_{j}|do(V_{i}=v_{i}\text{\&}\vec{S}=\vec{s}\prime ))\neq P(V_{j}=v_{j}|do(V_{i}=v_{i}\prime \&\vec{S}=\vec{s}\prime ))$. That is, an arrow is drawn from *V_i_* to *V_j_* just in case there is some assignment of values to all other variables in V such that the value of *V_i_* makes a difference to the probability distribution over the values of *V_j_* when the other variables in V take the assigned values. As in the deterministic case, where there is an arrow from *V_i_* to *V_j_*, *V_i_* is said to be a parent of *V_j_*, and *V_j_* to be a child of *V_i_*. Once again, ancestorhood is defined in terms of the transitive closure of parenthood, and descendanthood in terms of the transitive closure of childhood.

The result of applying this convention to the model of our probabilistic preemption scenario is, once again (and not by accident), the graph given as Figure 1 (in Section 5, above). Previously, a directed edge from a variable *V_i_* to a variable *V_j_* represented the fact that there is some pair of possible values $\left\{v_{i},v_{i}^{\prime }\right\}$ ($v_{i}\neq v_{i}^{\prime }$) of *V_i_*, some pair of possible values $\left\{v_{j},v_{j}^{\prime }\right\}$ ($v_{j}\neq v_{j}^{\prime }$) of *V_j_*, and some assignment $\vec{S}=\vec{s}\prime$ of values to the variables $\vec{S}=V\setminus \{V_{i},V_{j}\}$ such that if we held $\vec{S}$ fixed at $\vec{S}=\vec{s}\prime$ by interventions, then an intervention to set *V_i_* = *v_i_* would result in *V_j_* = *v_j_*, while an intervention to set $V_{i}=v_{i}\prime$ would result in $V_{j}=v_{j}^{\prime }$. Now it represents the fact that there is a pair of possible values, $\left\{v_{i},v_{i}^{\prime }\right\}$ ($v_{i}\neq v_{i}^{\prime }$) of *V_i_*, some possible value *v_j_* of *V_j_*, and some assignment $\vec{S}=\vec{s}\prime$ of values to the variables $\vec{S}=V\setminus \{V_{i},V_{j}\}$ such that if we held fixed $\vec{S}=\vec{s}\prime$ by interventions, then the probability of *V_j_* = *v_j_* would be different depending on whether we intervened to set *V_i_* = *v_i_* or $V_{i}=v_{i}\prime$. As seen in Section 6, above, the former case is arguably just a special case of the latter, namely, a case in which the probability of *V_j_* = *v_j_* would be one if we intervened to set *V_i_* = *v_i_*, but zero if we intervened to set $V_{i}=v_{i}^{\prime }$ (while holding fixed, by interventions, $\vec{S}=\vec{s}\prime$).

I have so far implicitly been supposing that a probabilistic causal model, $M^{*}=\langle V,P,do(\cdot )\rangle$, summarizes a set of counterfactuals about what the probability distribution over V would have been if any subset, $\vec{V}\prime$, of the variables in V had taken any possible set of values $\vec{V}\prime =\vec{v}\prime$. These counterfactuals are expressed by formulas of the form $P(\cdot |do(\vec{V}\prime =\vec{v}\prime ))$. Indeed, construing do(·) as a function that takes a probability distribution and a formula of the form $\vec{V}\prime =\vec{v}\prime$ as inputs and yields a counterfactual probability distribution $P(\cdot |do(\vec{V}\prime =\vec{v}\prime ))$ as an output, I suggested that a model $M^{*}=\langle V,P,do(\cdot )\rangle$ is appropriate only if, where *P* and $\vec{V}\prime =\vec{v}\prime$ are the inputs to do(·), the outputted distribution $P(\cdot |do(\vec{V}\prime =\vec{v}\prime ))$ is the chance distribution that truly would obtain if it had been that $\vec{V}\prime =\vec{v}\prime$. This, as I suggested, is analogous to Hitchcock’s requirement that an appropriate deterministic SEM ‘entail no false counterfactuals’ (Hitchcock [2001a], p. 287). Both requirements commit the requirer to a semantics for counterfactuals that is independent of the model in question. As suggested in the earlier discussion of deterministic SEMs, a semantics along the lines of those given by Lewis ([1979]) or Woodward ([2005]) would fill the bill.

Still, as discussed in Section 3, it is possible to regard deterministic SEMs as representing causal mechanisms, which are taken as primitive rather than as simply summarizing counterfactuals. The same is true of probabilistic causal models. On this view, a probabilistic causal model is construed as an ordered triple, 〈V,P,G〉, where (as before) V is a set of variables and *P* is a probability distribution defined on the field of events generated by those variables, but where G is a graph with the variables in V as its nodes. On this approach, it is typically required that the pair 〈P,G〉 obey the causal Markov condition (CMC) (Spirtes *et al.* [2000], pp. 29–30): each variable, V∈V, is probabilistically independent of its non-descendants given the values of its parents (where the variables that count as descendants of *V* and those that count as parents of *V* are evaluated with respect to G). The edges in G are taken to represent causal mechanisms, interventions are defined (contra Woodward [2005], p. 98) in terms of manipulations of G (Spirtes *et al.* [2000], pp. 47–53), and a semantics for counterfactuals (whose consequents concern the probabilities of primitive events or Boolean combinations of primitive events in the field generated by the variables in V) is given (with the aid of the CMC) in terms of these manipulations of G (Spirtes *et al.* [2000], pp. 47–53). As Woodward puts it, this alternative approach ‘defines the notion of an intervention with respect to the correct causal graph for the system in which the intervention occurs’ (Woodward [2005], p. 110). Consequently, Woodward points out, unlike his own approach, it does not ‘give us a notion of intervention that can be used to provide an interpretation for what it is for such a graph to be correct’ (Woodward [2005], p. 110).

In other words, this alternative approach, which construes a probabilistic causal model as a triple 〈V,P,G〉; takes a (causal-mechanism-representing) graph, G, as basic; and seeks to define in terms of G (with the help of the assumption that the CMC is satisfied by the pair 〈P,G〉) a function—which can be denoted do(·) and called an ‘intervention’—that takes the probability distribution *P* and any conjunction, $\vec{V}\prime =\vec{v}\prime$, of primitive events in the field generated by V as inputs, and yields as an output a new probability distribution, $P(\cdot |do(\vec{V}\prime =\vec{v}\prime ))$. The ‘summaries of counterfactuals’ view, by contrast, construes a probabilistic causal model as an ordered triple, 〈V,P,do(·)〉, thus taking the function do(·)—which takes a probability distribution and a conjunction, $\vec{V}\prime =\vec{v}\prime$, of primitive events in the field generated by V as inputs, and yields as an output a new probability distribution $P(\cdot |do(\vec{V}\prime =\vec{v}\prime ))$—as a primitive, and takes it as a requirement of appropriateness that (when *P* and $\vec{V}\prime =\vec{v}\prime$ are the inputs) the outputted distribution $P(\cdot |do(\vec{V}\prime =\vec{v}\prime ))$ is the chance distribution that truly would obtain if the variables $\vec{V}\prime$ were set to the values $\vec{V}\prime =\vec{v}\prime$ by interventions (where now the notion of an intervention is taken to be independently defined; see Woodward [2005], p. 98) or, alternatively, by small miracles. A correct graphical representation of the model can then be given in accordance with the conventions described above.

If probabilistic causal models are taken to summarize counterfactuals (in this case, counterfactuals about probabilities), then the possibility of giving a reductive account of actual causation in terms of probabilistic causal models is retained. But the account will be reductive only if the counterfactuals are given a semantics (perhaps along the lines of that given by Lewis ([1986b]), Postscript D) that does not appeal to causal notions. It will not be fully reductive if the counterfactuals are given a semantics that appeals to causal notions, such as Woodward’s notion of an intervention (Woodward [2005], p. 98). But even in that case, it may still be illuminating for the reasons that were discussed in Section 3 in connection with analyses of actual causation in terms of deterministic SEMs that are taken to summarize interventionist counterfactuals. Similarly, if probabilistic causal models are instead taken to have a graph representing causal mechanisms among their primitives, then analyses of actual causation in terms of probabilistic causal models may be illuminating for much the same reasons as analyses of actual causation in terms of deterministic SEMs are illuminating, even where structural equations are construed as representing causal mechanisms. But they will not be fully reductive. The analysis of probabilistic actual causation to be advanced in the next section is compatible with either of these views of probabilistic causal models.

## 8 A Proposed Probabilistic Extension of Halpern and Pearl’s Definition

With the notion of a probabilistic causal model in place, as discussed in the previous section, we are now in a position to modify Halpern and Pearl’s definition so that it can handle probabilistic preemption. Specifically, suppose that $M^{*}$ is a probabilistic causal model and that $\vec{u}$ is the actual context, that is, it is the set of values that the exogenous variables in $M^{*}$ have in the actual world (or, more generally, the world of evaluation). The analysis that I wish to propose as the natural extension of Halpern and Pearl’s definition to the case of probabilistic actual causation is PC^46^^,^^47^^,^^48^:

PC: $\vec{X}=\vec{x}$ is an *actual cause of*ϕ*in*$\left(M^{*},\vec{u}\right)$ (that is, in model $M^{*}$ given context $\vec{u}$) if and only if the following three conditions hold:

PC1. Both $\vec{X}=\vec{x}$ and ϕ are true in the actual world (or, more generally, the world of evaluation).
PC2. There exists a partition, $\left(\vec{Z},\vec{W}\right)$, of Y (that is, the set of endogenous variables in the model $M^{*}$), with $\vec{X}\subseteq \vec{Z}$ and some setting $\left(\vec{x}\prime ,\vec{w}\prime \right)$ of the variables in $\left(\vec{X},\vec{W}\right)$, such that where in the actual world $Z_{i}=z_{i}^{*}$ for all $Z_{i}\in \vec{Z}$, the following holds:
  - $P(\phi |do(\vec{X}=\vec{x}\text{\&}\vec{W}=\vec{w}\prime ))>P(\phi |do(\vec{X}=\vec{x}\prime \text{\&}\vec{W}=\vec{w}\prime ))$. In words, if the variables in $\vec{W}$ had taken the values $\vec{W}=\vec{w}\prime$, then the probability of ϕ would be higher if the variables in $\vec{X}$ took the values $\vec{X}=\vec{x}$ than if the variables in $\vec{X}$ took the values $\vec{X}=\vec{x}\prime$.
  - $P(\phi |do(\vec{X}=\vec{x}\text{\&}\vec{W}=\vec{w}\prime \text{\&}\vec{Z}\prime ={\vec{z}}^{*}))>P(\phi |do(\vec{X}=\vec{x}\prime \text{\&}\vec{W}=\vec{w}\prime ))$ for all subsets $\vec{Z}\prime$ of $\vec{Z}$. In words, if the variables in $\vec{W}$ had taken the values $\vec{W}=\vec{w}\prime$, and the variables in $\vec{X}$ had taken the values $\vec{X}=\vec{x}$, and all of the variables in an arbitrary subset of $\vec{Z}$ had taken their actual values, then the probability of ϕ would still have been higher than if the variables in $\vec{W}$ had taken the values $\vec{W}=\vec{w}\prime$ and the variables in $\vec{X}$ had taken the values $\vec{X}=\vec{x}\prime$.
PC3. $\vec{X}$ is minimal; no strict subset $\vec{X}\prime$ of $\vec{X}$ is such that if $\vec{X}$ is replaced by $\vec{X}\prime$ in PC2, then no change to the values of the counterfactual probabilities that are appealed to in PC2 results. Minimality ensures that only those elements of the conjunction $\vec{X}=\vec{x}$ that are relevant to the probabilities of ϕ appealed to in PC2 are considered part of a cause; inessential elements are pruned.

In the probabilistic preemption case described in Section 6 above, PC correctly counts *C* = 1 as an actual cause of *D* = 1. To see this, note that the actual context (that is, the set of actual values of the exogenous variables) is simply $\vec{u}=\{CI=1,BI=1\}$. Let $\vec{X}=\{C\}$, with $\vec{x}=\{C=1\}$ and $\vec{x}\prime =\{C=0\}$. Let ϕ be *D* = 1. In the actual world, *C* = 1 and *D* = 1, so condition PC1 is satisfied. If PC2 is satisfied, then PC3 will also be satisfied because $\vec{X}=\{C\}$ has no (non-empty) subsets; and if PC2(a) is satisfied, then this implies that, in the circumstances $\vec{W}=\vec{w}\prime$, the values of the variables in $\vec{X}=\{C\}$ make a difference to the probability of ϕ. So everything hinges on whether PC2 is satisfied.

To see that PC2 is satisfied, let $\vec{Z}=\langle C,S,D\rangle$, let $\vec{W}=\langle B,T\rangle$, and let $\vec{w}\prime =\{B=1,T=0\}$. First note that PC2(a) is satisfied because:

(8) P(D=1|do(C=1&B=1&T=0))>P(D=1|do(C=0&B=1&T=0)).

In words, the probability that McCluskey would have died if Corleone had issued his order, Barzini had issued his order, but Turk hadn’t shot is greater than the probability that McCluskey would have died if Corleone had not issued his order, Barzini had issued his order, but Turk hadn’t shot. In fact, given the stipulations of the example, the former probability is approximately 0.45, while the latter is approximately 0. It is important to bear in mind here the non-backtracking nature of the counterfactuals. In particular, the probabilities are those that would obtain if Turk’s not shooting were brought about by an intervention, small miracle, or local surgery that does not affect whether or not Sonny shoots. This is what is indicated by the do(·) operator.

To see that PC2(b) is satisfied, note that if it had been the case that *C* = 1, *B* = 1, and *T* = 0, then the probability of *D* = 1 would have been higher, even if *S* had taken its actual value *S* = 1, than it would have been if *C* = 0, *B* = 1, and *T* = 0. That is,

(9) P(D=1|do(C=1&B=1&T=0&S=1))>P(D=1|do(C=0&B=1&T=0)).

In words, if Barzini had issued his order but Turk hadn’t shot, then the probability of McCluskey’s death would have been higher if Corleone issued his order even if Sonny had shot, than it would have been if Corleone hadn’t issued his order. Indeed, given the stipulations of the example, the former probability is approximately 0.5, while the latter is approximately 0.^49^

So PC2(b) is satisfied. We have already seen that PC1 and PC2(a) are satisfied, and that PC3 is satisfied if PC2 is. Consequently, PC yields the correct verdict that *C* = 1 is an actual (probabilistic) cause of *D* = 1.

PC also yields the intuitive verdict that *B* = 1 (Barzini’s order) is not an actual cause of *D* = 1. In order to get the sort of contingent probabilistic dependence of *D* = 1 upon *B* = 1 required by condition PC2(a), it will be necessary to include in the antecedents of the relevant counterfactuals the fact that at least one variable on the Corleone process—that is, either *C* or *S*—takes (the non-actual value) 0. The trouble is that, in such circumstances, if *B* and *T* took their actual values, *B* = 1 and *T* = 0, then the probability of *D* = 1 would be no higher than if *B* took the value *B* = 0. This is contrary to the requirement of condition PC2(b).

For example, consider the obvious partition $\vec{Z}=\langle B,T,D\rangle$ and $\vec{W}=\langle C,S\rangle$, and consider the assignment $\vec{w}\prime =\langle C=1,S=0\rangle$. Condition PC2(a) is satisfied for this partition and this assignment. In particular, it is true that

(10) P(D=1|do(B=1&C=1&S=0))>P(D=1|do(B=0&C=1&S=0)).

That is to say, in circumstances in which Corleone issues his order but Sonny doesn’t shoot, the probability of McCluskey’s dying would be higher if Barzini issued his order than if Barzini didn’t issue his order. Given the stipulations of our example, the former probability is approximately 0.81, while the latter is approximately 0.

But notice that PC2(b) is not satisfied for this partition and assignment of values to $\vec{W}$. For take $\vec{Z}\prime =\{T\}\subset \vec{Z}$, and observe that

(11) P(D=1|do(B=1&C=1&S=0&T=0))≤P(D=1|do(B=0&C=1&S=0)).

That is to say, in circumstances in which Corleone issued his order but Sonny didn’t shoot, if (as was actually the case) Barzini issued his order, but Turk didn’t shoot, the probability of McCluskey’s death would have been no higher than it would have been if Barzini hadn’t issued his order in the first place. Intuitively, this is because, in circumstances where Corleone issues his order but Sonny doesn’t shoot, Barzini’s order only raises the probability of McCluskey’s death because it raises the probability of Turk’s shooting. So (in circumstances in which Corleone issues his order but Sonny doesn’t shoot), the probability of McCluskey’s death if Barzini had issued his order but Turk had not shot would have been no higher than if (in the same circumstances) Barzini simply hadn’t issued his order.

Nor is there any other partition, $\left(\vec{Z},\vec{W}\right)$, of the endogenous variables {C,B,S,T,D} such that PC2 is satisfied. In particular, none of the remaining variables on the Barzini process, {*T*, *D*}, can be assigned to $\vec{W}$ instead of $\vec{Z}$ if PC2(a) is to be satisfied, for the values of each of these variables screens off *B* from *D*, so the result would be that PC2(a) wouldn’t hold for any assignment, $\vec{w}\prime$, of values to variables in $\vec{W}$. On the other hand, reassigning all or some of the variables on the initial Corleone process, {*C*, *S*}, to $\vec{Z}$ will not affect the fact that PC2(b) fails to obtain. This is because no matter what subset of {*C*, *S*} we take $\vec{W}$ to comprise, and no matter what values $\vec{w}\prime$ are assigned to that subset by interventions, the probabilistic relevance of *B* to *D* remains entirely by way of its relevance to *T*. So it will remain true that where $\vec{W}=\vec{w}\prime$, if *B* = 1 and *T* = 0, then the probability of *D* = 1 would be no higher than if *B* = 0, in violation of PC2(b). (Again, it is important to remember that the relevant worlds where $\vec{W}=\vec{w}\prime$ and *B* = 1 and *T* = 0 hold are those in which *T* has the value *T* = 0 as the result of an intervention or similar, rather than *T*’s value being influenced in the usual way by the value of *S*.)

So PC gives the correct diagnosis of probabilistic preemption. It does so on intuitively the correct grounds. Specifically, the reason that Corleone’s order is counted as a cause is that (i) given Turk’s non-shooting, Corleone’s order raised the probability of McCluskey’s death; and (ii) there is a complete causal process running from Corleone’s order to McCluskey’s death. This is indicated by the fact that for arbitrary subsets of events on the Corleone process, it is true that (in circumstances in which Turk doesn't shoot), if Corleone had issued his order and the variables representing those events had taken their actual values, then the probability of McCluskey’s death would have remained higher than if Corleone had never issued his order in the first place.

By contrast, Barzini’s order isn’t counted as a cause because, although (i) given Sonny’s non-shooting, Barzini’s order would raise the probability of McCluskey’s death; nevertheless, (ii) there is no complete causal process from Barzini’s order to McCluskey’s death as indicated by the fact that if Barzini had issued his order and Sonny hadn’t shot but (as was actually the case) Turk didn’t shoot, then the probability of McCluskey’s death would have been no higher than it would have been if (Sonny hadn’t shot and) Barzini hadn’t issued his order in the first place.

It was noted above that Halpern and Pearl ([2005], p. 859) suggest that their definition AC might reasonably be adjusted in light of the contrastive nature of many causal claims. Indeed, as noted above, several philosophers have argued rather convincingly that actual causation is contrastive in nature (for example, Hitchcock [1996a], [1996b]; Schaffer [2005], [2013]), and specifically that causation is a quaternary relation, with the cause, the effect, a set of alternatives to the cause, and a set of alternatives to the effect as its relata. In the present context, this would mean that the primary analysandum is not ‘$\vec{X}=\vec{x}$ is an actual cause of ϕ’, but rather ‘$\vec{X}=\vec{x}$ rather than $\vec{X}=\vec{x}\prime$ is an actual cause of ϕ rather than ϕ′’, where $\vec{X}=\vec{x}\prime$ denotes a set of formulas of the form $\vec{X}=\vec{x}\prime$, such that for each such formula $\vec{x}\neq \vec{x}\prime$, and where ϕ′ represents a set of formulas of the form ϕ′, such that for each such formula, ϕ is incompatible with ϕ′.

The case for turning PC into an analysis of a four-place relation is just as compelling as the case for the corresponding modification of AC. As it stands, where the cause and/or effect variables are multi-valued, PC (just like the unmodified AC) is liable to run into difficulties. Consider a case where Doctor can administer no dose, one dose, or two doses of medicine to Patient. Let *M* be a variable that takes value *M* = 0 if no dose is administered, *M* = 1 if one dose is administered, and *M* = 2 if two doses are administered. Suppose that Patient will recover with chance 0.1 if no dose is administered, with chance 0.9 if one dose is administered, and with chance 0.5 if two doses are administered (two doses is an ‘overdose’, which would adversely affect Patient’s natural immune response). Let *R* be a variable that takes value *R* = 1 if Patient recovers and *R* = 0 if she does not. Suppose that the context is such that Doctor is equally disposed to each of the three courses of action. We can represent the (exogenous) intentions of Doctor that give rise to this disposition using a (exogenous) variable, *D*, that takes value *D* = 1 if Doctor has these intentions and *D* = 0 if she does not. Suppose that Doctor in fact administers two doses of medicine, and Patient recovers.

Did Doctor’s administering two doses of medicine cause Patient to recover? I think the natural reaction is one of ambivalence. After all, while it is true that Patient’s recovery was more likely given that Doctor administered two doses than it would have been if she had administered zero doses, it was less likely than if Doctor had administered one dose. If we focus on the fact that Doctor could have administered just one dose, we might be inclined to say that Patient recovered despite Doctor’s action. If we focus on the fact that Doctor could have administered zero doses, we might be inclined to say that Patient recovered because of Doctor’s action. One plausible interpretation of our ambivalent attitude is that actual causation is contrastive in nature, and ‘Doctor’s administering two doses of Medicine caused Patient to recover’ is ambiguous between ‘Doctor’s administering two doses of Medicine rather than no doses caused Patient to recover’ (to which most people would presumably assent) and ‘Doctor’s administering two doses of Medicine rather than one dose caused Patient to recover’ (to which most people would presumably not assent).

Yet, as it stands, PC delivers the unequivocal result that Doctor’s action (*M* = 2) was an actual cause of Patient’s recovery (*R* = 1), where the variable set for our model is {*D*, *M*, *R*}. To see this, let $\vec{X}=\{M\}$, let $\vec{x}=\{M=2\}$, and let ϕ be *R* = 1. Consider the partition $\left(\vec{Z},\vec{W}\right)$ of the endogenous variables such that $\vec{Z}=\langle M,R\rangle$ and $\vec{W}=Ø$. Condition PC1 is satisfied because *M* = 2 and *R* = 1 are the actual values of *M* and *R* (or rather the values that obtain in the world in which our causal scenario plays out). If condition PC2 is satisfied, then condition PC3 is satisfied because if PC2(a) is satisfied, then this implies that (in the relevant circumstances) the value of *M* makes a probabilistic difference to that of *R*, and there are no (non-empty) subsets of {*M*}. Condition PC2(a) is satisfied because it requires only that there be one alternative value of *M* such that if *M* took that value (and the variables in $\vec{W}$ took some possible assignment $\vec{W}=\vec{w}$—something that trivially holds because there are no variables in $\vec{W}$ in this case),^50^ then the probability of *R* = 1 would be lower than if *M* had taken *M* = 2. In this case, *M* = 0 is such a value. So PC2(a) is satisfied. Condition PC2(b) is rather trivially satisfied: since there are no variables in $\vec{Z}\setminus M,R$, PC2(b) just reduces to the requirement that if *M* had taken the value *M* = 2, then the probability of *R* = 1 would have been higher than it would have been if *M* had taken the value *M* = 0, which clearly holds in the example given. So PC2 is satisfied. We have already seen that PC1 is satisfied, and that PC3 is satisfied if PC2 is satisfied. Consequently, as it stands, PC implies that Doctor’s action (*M* = 2) was an actual cause of Patient’s recovery (*R* = 1).

The unequivocal nature of PC’s verdict contrasts with the verdict of intuition, which is equivocal. Thus, as was the case with AC, it would seem desirable to modify PC so that it can capture the nuances of our contrastive causal judgements. This is easily achieved. To turn PC into an analysis of $\vec{X}=\vec{x}$ rather than $\vec{X}=\vec{x}\prime$ being an actual cause of ϕ, we simply need to require that PC2 hold not just for some non-actual setting of $\vec{X}$, but for precisely the setting $\vec{X}=\vec{x}\prime$.

This revised version of PC yields the intuitively correct verdict that *M* = 2 rather than *M* = 0 was an actual cause of *R* = 1. Specifically, taking the relevant contrast to *M* = 2 to be *M* = 0, the revised version of PC is satisfied for precisely the same reason that taking $\vec{X}=\vec{x}\prime$ to be *M* = 0 showed the original version of PC to be satisfied. The revised version of PC also yields the verdict that *M* = 2 rather than *M* = 1 is not a cause of *R* = 1. This is because the revised version of PC2(a) is violated when we take *M* = 1 to be the contrast to *M* = 2. This is because it’s not the case that if *M* had taken the value *M* = 2, then the probability that *R* would have taken *R* = 1 would have been higher than it would have been if *M* had taken the value *M* = 1 (in fact it would have been lower in the example given). So the revised PC yields the desired verdicts about these contrastive causal claims. Indeed, the revised PC can explain the equivocality of intuition about the claim ‘*M* = 2 was an actual cause of *R* = 1’ in terms of its ambiguity between ‘*M* = 2 rather than *M* = 0 was an actual cause of *R* = 1’ (which it evaluates as true) and ‘*M* = 2 rather than *M* = 1 was an actual cause of *R* = 1’ (which it evaluates as false).

More generally, to turn PC into an analysis of ‘$\vec{X}=\vec{x}$ rather than $\vec{X}=\vec{x}\prime$ is an actual cause of ϕ’, where $\vec{X}=\vec{x}\prime$ denotes a set of formulas of the form $\vec{X}=\vec{x}\prime$, we simply need to require that PC2 hold for every event of the form $\vec{X}=\vec{x}\prime$ in $\vec{X}=\vec{x}\prime$. This extension to allow for a possibly non-singleton contrast set $\vec{X}=\vec{x}\prime$ is particularly valuable when the putative cause variable is many valued, or even continuous.

As an illustration, suppose that Driver is driving at 50 miles per hour (mph) and crashes. Let *S* be a variable representing Driver’s speed in mph and let *C* be a variable where *C* = 1 if she crashes, and *C* = 0 if not. Suppose that *B* is an exogenous variable that represents the (exogenous) dispositions of the driver, upon which her speed can be taken to depend. Suppose (for simplicity) that the probability of Driver’s crashing is a strictly increasing function of her speed, $P(C=1)=f_{P(C=1)}(S)$. Was Driver’s driving at 50 mph an actual cause of her crash? I think that it’s natural to feel ambivalent. There seems to me to be a strong temptation to say: ‘Driver’s driving at 50 mph rather than less than 50 mph was a cause of her crash’ but that ‘Driver’s driving at 50 mph rather than more than 50 mph was not a cause of her crash’. (We might feel that it is appropriate to say that ‘Driver crashed despite driving at 50 mph rather than more than 50 mph’.)

The revised version of PC, which allows for (non-singleton) contrast sets, can capture these intuitions. It vindicates the assertion that Driver’s driving at 50 mph rather than less than 50 mph was a cause of her crash. In this case, the ‘rather than’ clause indicates that the contrast set is to be taken as the set of all those possible values of *S* that are less than 50, that is, the set {x:x∈R(S),x<50}, where R(S) denotes the range of *S* (that is, the set of all of *S*’s possible values). Suppose that our model has the variable set {*B*, *S*, *C*}. Let $\vec{X}=\{S\}$, let $\vec{x}=\{S=50\}$, let ϕ be *C* = 1, and let the partition ($\vec{Z},\;\vec{W}$) of the endogenous variables be the partition such that $\vec{Z}=\langle S,C\rangle$ and $\vec{W}=Ø$. Condition PC1 is satisfied because *S* = 50 and *C* = 1 in the world in question. Condition PC3 is satisfied if revised condition PC2 is satisfied because the satisfaction of the revised PC2(a) implies that *S* = 50 makes a difference (in the relevant circumstances, and relative to the appropriate contrast set) to the probability that *C* = 1, and because there are no (non-empty) subsets of {*S*}. Revised condition PC2(a) is satisfied because it is true that if *S* had taken *S* = 50, as it actually did, then the probability of *C* = 1 would have been higher than it would have been if *S* had taken any of the values in the set {x:x∈R(S),x<50}. Revised condition PC2(b) is satisfied rather trivially because there are no variables in $\vec{Z}\setminus S,C$. So the revised PC2(b) just reduces to the requirement that if *S* had taken the value *S* = 50, then the probability of *C* = 1 would have been higher than if *S* had taken any value less than 50. It was a stipulation of the example that this is the case. So PC2 is satisfied. We have already seen that PC1 is satisfied, and that PC3 is satisfied if PC2 is. So the revised version of PC yields the intuitively correct result that *S* = 50, rather than *S* < 50, was a cause of *C* = 1.

The revised version of PC also vindicates the intuition that Driver’s driving at 50 mph rather than more than 50 mph was not a cause of her crash. In this case, the ‘rather than’ clause indicates that the contrast set is to be taken to be that containing all those values of *S* that are greater than 50, that is, {y:y∈R(S),y>50}. We can again take our model to have the variable set {*B*, *S*, *C*}, and we can again let $\vec{X}=\{S\},\;\vec{x}=\{S=50\}$, and let ϕ be *C* = 1. Again, condition PC1 is satisfied because *S* = 50 and *C* = 1 in the world in question, and condition PC3 is satisfied if PC2 is, for the same reasons as before. But, since the probability of *C* = 1 is not higher given *S* = 50 than it would have been if *S* had taken any of the values in the set {y:y∈R(S),y>50} (even if the variables in $\vec{W}$—of which there are none—had taken some set of possible values), the revised PC2(a) is not satisfied. The revised version of PC therefore yields the intuitively correct result that *S* = 50, rather than *S* > 50, was not a cause of *C* = 1.

So the revised PC captures our intuitive judgements concerning contrastive causal claims in this case.^51^ It also allows an explanation of why we feel ambivalent about the claim that ‘Driver’s driving at 50 mph was a cause of her crash’. The explanation is that this causal claim is incomplete, since no contrast sets are specified. As such, the revised PC doesn’t yield a verdict about whether this claim is true or false. In particular, the claim is ambiguous between ‘Driver’s driving at 50 mph rather than less than 50 mph caused her crash’ (which the revised PC evaluates as true) and ‘Driver’s driving at 50 mph rather than more than 50 mph caused her crash’ (which it evaluates as false).^52^

We have seen that building contrast into PC on the cause side allows it to better capture our intuitions. We may find it plausible to build contrast in on the effect side too. To change our earlier example involving Doctor and Patient somewhat, suppose (for simplicity) that Doctor only has two options: to administer no dose of medicine (*M* = 0) or to administer one dose of medicine (*M* = 1). In this case, the variable *M* is thus binary. On the other hand, suppose this time that the recovery variable *R* has three possible values: *R* = 0 if Patient fails to recover, *R* = 1 if she recovers speedily, and *R* = 2 if she recovers slowly. Suppose, moreover, that the probability distributions over the various values of *R* that would result from the various values of *M* are those given in Table 1, where the probability values given are those that would result for the various values of *R* specified in the top row if *M* had taken the various values specified in the leftmost column.

**Table 1.** The probability value given in each cell, *c*, of the table is that which would obtain for the value of *R*, specified at the top of the column that *c* occupies, if *M* had taken the value specified at the left of the row that *c* occupies

	*R* = 0	*R* = 1	*R* = 2
*M* = 0	0.1	0.1	0.8
*M* = 1	0.1	0.8	0.1

Suppose this time that Doctor in fact administers zero doses of medicine (*M* = 0), and that Patient recovers slowly (*R* = 2). We may well feel inclined to judge it to be false that Doctor’s administering zero doses rather than one dose caused Patient to recover slowly rather than not recovering at all, but true that Doctor’s administering zero doses rather than one dose caused Patient to recover slowly rather than quickly. After all, Doctor’s administering zero doses made no difference to the probability of Patient’s not recovering. However, it did make a difference to the probability of Patient’s recovering quickly.

Analysis PC can be extended to achieve this result. Adapting a suggestion due to Schaffer ([2005], p. 348), I suggest that in order to analyse a claim of the form ‘$\vec{X}=\vec{x}$ rather than $\vec{X}=\vec{x}\prime$ actually caused ϕ rather than ϕ′’, we simply need to (i) require that PC2 hold, not just for some non-actual setting of $\vec{X}$, but for precisely the setting $\vec{X}=\vec{x}\prime$ (as discussed above); and (ii) add the requirement that the resulting PC2(a) is also satisfied when we replace $\vec{X}=\vec{x}$ with $\vec{X}=\vec{x}\prime$ and vice versa, and replace ϕ with ϕ′ throughout. The upshot of all of this is that the modified analysis requires not only that (in the circumstances that $\vec{W}=\vec{w}\prime$) the probability of ϕ is higher in the presence of $\vec{X}=\vec{x}$ than in the presence of $\vec{X}=\vec{x}\prime$, but also that the probability of the alternative ϕ′ would be higher in the presence of $\vec{X}=\vec{x}\prime$ than in the presence of $\vec{X}=\vec{x}$.

This handles the present example. Suppose the endogenous variables in our model just to be *M* and *R*, and let the partition ($\vec{Z},\;\vec{W}$) be the one such that $\vec{Z}=\langle M,R\rangle$ and $\vec{W}=Ø$. We get the correct result that *M* = 0, rather than *M* = 1, was an actual cause of *R* = 2, rather than *R* = 1: the probability of *R* = 2 would be higher if *M* took the value *M* = 0 than it would be if *M* took the value *M* = 1; and the probability of *R* = 1 would be higher if *M* took the value *M* = 1 than it would be if *M* took the value *M* = 0. We also get the correct result that *M* = 0 rather than *M* = 1 did not cause *R* = 2 rather than *R* = 0 because while the probability of *R* = 2 would be higher if *M* took the value *M* = 0 than it would be if *M* took the value *M* = 1, it is not the case that the probability of *R* = 0 would be higher if *M* took the value *M* = 1 than it would be if *M* took the value *M* = 0.

More generally, suppose that we wish to analyse claims of the form ‘$\vec{X}=\vec{x}$ rather than $\vec{X}=\vec{x}\prime$ was an actual cause of ϕ rather than ϕ′’, where $\vec{X}=\vec{x}\prime$ denotes a set of formulas of the form $\vec{X}=\vec{x}\prime$ such that for each such formula, $\vec{x}\neq \vec{x}\prime$, and where ϕ′ represents a set of formulas of the form ϕ′ such that for each such formula, ϕ is incompatible with ϕ′. Then (again adapting a proposal due to Schaffer [2005], p. 348) we need to require that for each event of the form $\vec{X}=\vec{x}\prime$ in $\vec{X}=\vec{x}\prime$, (i) PC2 holds, not just for some non-actual setting of $\vec{X}$, but for precisely the setting $\vec{X}=\vec{x}\prime$; and (ii) there is some ϕ′∈ ϕ′ such that PC2(a) also holds when we replace $\vec{X}=\vec{x}$ with the specific setting $\vec{X}=\vec{x}\prime$ and vice versa, and replace ϕ with ϕ′ throughout.^53^ The upshot of all of this will be that the modified analysis requires not only that (in the circumstances $\vec{W}=\vec{w}\prime$) the probability of ϕ is higher in the presence of $\vec{X}=\vec{x}$ than it is in the presence of any formula of the form $\vec{X}=\vec{x}\prime$ in $\vec{X}=\vec{x}\prime$, but also that each formula of the form $\vec{X}=\vec{x}\prime$ in $\vec{X}=\vec{x}\prime$ makes one of the alternatives ϕ′ in ϕ′ to ϕ more likely than does $\vec{X}=\vec{x}$.

This revised definition reduces to the original PC where the putative cause is a primitive event (rather than a conjunction of primitive events), where the putative effect is a primitive event (rather than a Boolean combination of primitive events) and where the variables representing cause and effect are binary. This was the case in our probabilistic preemption scenario. For instance, consider the actual causal relation between *C* = 1 (Corleone’s order) and *D* = 1 (McCluskey’s death). In this case, there is only one non-actual possible value of the cause variable—namely, *C* = 0. This means that the non-actual setting of the cause variable, $\vec{X}=\vec{x}\prime$, appealed to in unrevised condition PC2, can only be *C* = 0. There is also only one non-actual possible value of the effect variable—namely, *D* = 0. This means that the fact that *C* = 1 raised the probability of *D* = 1 (in the specified circumstances, in which *T* = 0) automatically implies that *C* = 0 raised the probability of *D* = 0 (in those same circumstances). Consequently, in this case, saying that *C* = 1 is an actual cause of *D* = 1 is effectively equivalent to saying that *C* = 1 rather than *C* = 0 is an actual cause of *D* = 1 rather than *D* = 0.

In closing this section, it is worth noting that while the causal notion upon which (following Halpern and Pearl [2001], [2005]) I have been focusing here is that of actual causation, I think that other causal notions can be fruitfully analysed within the present framework. I’m inclined to think that in the probabilistic case, just as in the deterministic case, prevention is just the flip-side of actual causation: if $\vec{X}=\vec{x}$ (rather than $\vec{X}=\vec{x}\prime$) is an actual cause of ϕ rather than ϕ′, then $\vec{X}=\vec{x}$ (rather than $\vec{X}=\vec{x}\prime$) prevents ϕ′ rather than ϕ from happening.

There are other notions in the vicinity, such as ‘negative causal relevance’. For example, concerning the driving case described above, we might well be inclined to say that Driver’s driving at 50 mph, rather than over 50 mph, was negatively causally relevant to the crash. The notion of negative causal relevance seems to be different from the notion of prevention. It would be clearly contradictory to say that $\vec{X}=\vec{x}$ prevented ϕ,^54^ but nevertheless ϕ obtained. But it is not obviously contradictory to say that $\vec{X}=\vec{x}$ was negatively relevant to ϕ, but ϕ obtained. In such circumstances, we might say things like ‘ϕ obtained despite $\vec{X}=\vec{x}$’ (for example, ‘the driver crashed despite driving at 50 mph rather than over 50 mph’). Likewise, positive causal relevance seems to be different to actual causation. While it is contradictory to say that $\vec{X}=\vec{x}$ caused ϕ, but ϕ didn’t obtain, it does not seem contradictory to say that $\vec{X}=\vec{x}$ was positively relevant to ϕ, but ϕ didn’t obtain. In such cases, we might say things like ‘ϕ failed to occur despite $\vec{X}=\vec{x}$’.

I suspect that talk of positive causal relevance and negative causal relevance is less well-regimented than talk of causation and prevention. The use of SEMs and probabilistic causal models allows us to distinguish a variety of precise causal notions (cf. Hitchcock [2009], pp. 305–6, [2001b], pp. 369–74) between which (I suspect) talk of ‘positive causal relevance’ and negative causal relevance is ambiguous. In particular, in the probabilistic context, saying that $\vec{X}=\vec{x}$ is positively causally relevant to ϕ may (I think) mean any one of the following (and perhaps more besides): (a) $\vec{X}=\vec{x}$ raises the probability of ϕ (in a suitably non-backtracking way, such as that captured by Inequality (1) in Section 6, above); (b) $\vec{X}=\vec{x}$ raises the probability of ϕ along one or more causal pathways (that is, when variables on all other pathways are held fixed): essentially the notion that PC2(a) is designed to capture (cf. Hitchcock [2001b], pp. 373–4); (c) $\vec{X}=\vec{x}$ raises the probability of ϕ along a causal pathway that represents a process that is complete except possibly for the effect itself (which is essentially the notion that I take to be captured by the whole of PC, if one simply drops the requirement that ϕ hold); or (d) $\vec{X}=\vec{x}$ is an actual cause of ϕ (which is the notion that I take to be captured by the whole of PC).

Saying that $\vec{X}=\vec{x}$ is negatively causally relevant to ϕ may (I think) mean any one of the following (and perhaps more besides): (a') $\vec{X}=\vec{x}$ lowers the probability of ϕ (in a suitably non-backtracking way, such as that captured by Inequality (1) if we were to replace the ‘>’ with a ‘<’); (b′) $\vec{X}=\vec{x}$ lowers the probability of ϕ along one or more causal pathways (which would be captured by PC2(a) if we replaced the ‘>’ with a ‘<’); (c′) $\vec{X}=\vec{x}$ lowers the probability of ϕ (raises the probability of ¬ϕ) along a causal pathway representing a process that is complete except possibly that ϕ occurs (despite $\vec{X}=\vec{x}$) (which is essentially the notion that I take to be captured by the whole of PC, if we were to replace the ‘>’s with ‘<’s and drop the requirement that ϕ hold); (d′) $\vec{X}=\vec{x}$ prevents ϕ (which I take to be captured by the whole of PC if we were to replace the ‘ >’s with ‘ <’s and replace the requirement that ϕ hold with the requirement that ¬ϕ hold); or (e) $\vec{X}=\vec{x}$ lowers the probability of ϕ (raises the probability of ¬ϕ) along a causal pathway representing a process that is complete except that ϕ does occur (despite $\vec{X}=\vec{x}$) (which is essentially the notion that I take to be captured by the whole of PC, if we were to replace the ‘ >’s with ‘ <’s^55^).^56^^,^^57^

In the next section, I will compare my analysis of probabilistic actual causation, PC, to an analysis of probabilistic causation developed by Twardy and Korb ([2011]), which is similar in spirit to my own. One difference between the two accounts is that Twardy and Korb ([2011], p. 906) advance their analysis as an analysis of causal relevance, rather than actual causation. Although they don’t make this entirely explicit, I think the most natural reading of what Twardy and Korb ([2011], pp. 902, 906) say indicates that, on their construal of causal relevance, $\vec{X}=\vec{x}$ is causally relevant to ϕ just in case either (d) or (e) holds. That is, just in case $\vec{X}=\vec{x}$ is an actual cause of ϕ (a notion that—setting aside complications due to contrastivity—I take to be captured by PC) or $\vec{X}=\vec{x}$ lowers the probability of ϕ (raises the probability of ¬ϕ) along a causal pathway representing a process that is complete except that ϕ does occur (despite $\vec{X}=\vec{x}$) (which is essentially the notion that I take to be captured by the whole of PC, if we were to replace the ‘ >’s with ‘ <’s).

I have focused on actual causation, which has the occurrence of the putative effect event caused as a necessary condition (and, as a corollary, prevention, which has the non-occurrence of the prevented event as a necessary condition), not because I think that the present approach can't distinguish a number of interesting causal notions (it can!), but because, first, actual causation is one causal notion of particular interest. For example, actual causation is particularly central to scientific explanation (especially when contrasted with notions such as probability raising, or probability raising along a pathway, where it is not required that there be a complete causal process connecting the probability raiser to the probability raisee). Second and (presumably) relatedly, as I have suggested, our talk of ‘causation’ (and ‘prevention’) is (I think) better regimented than our use of other causal notions—such as ‘causal relevance’—thus making it possible to use our causal talk to triangulate to a particular causal notion that can be precisely defined in terms of causal models. Nevertheless, I am very sympathetic to those who use the causal modelling framework to distinguish other interesting causal notions. Indeed, I have indicated in the previous three paragraphs how I would go about analysing several such notions, including the one that Twardy and Korb ([2011], p. 906) call ‘causal relevance’.

## 9 Twardy and Korb’s Account

A similar project to my own—namely, that of extending deterministic structural equations accounts of causation to the probabilistic context—has recently been pursued (independently) by Twardy and Korb ([2011]). Their account has some similarities to mine (hopefully reflecting a ‘convergence to the truth’!), but also differs in important respects. These differences leave their account susceptible to counterexamples that mine avoids.

One difference (which I take to be unproblematic) is that Twardy and Korb’s analogue of my condition PC2(a) (and Halpern and Pearl’s AC2(a)) appeals to contingent probabilistic difference-making (that is, contingent probability raising or contingent probability lowering). So, in essence, their version of my PC2(a) can be arrived at just by replacing ‘>’ with ‘≠’. As they indicate (Twardy and Korb [2011], p. 906), this reflects the fact that they wish to analyse a somewhat broader notion than that of ‘actual causation’, namely, that of ‘causal relevance’.^58^ For reasons discussed at the end of the previous section, I am confining my attention to actual causation (and, as a corollary, prevention). It seems to me that contingent probability raising is the relation that we need to focus upon in analysing actual causation, while contingent probability lowering is important in the analysis of prevention. Twardy and Korb ([2011], p. 906) appear to agree that contingent probability lowering is the relation of relevance for analysing prevention. They (Twardy and Korb [2011], p. 906) suggest that contingent probability raising is of relevance to analysing ‘promotion’, though they do not make it entirely clear what they take the relation between ‘promotion’ and actual causation to be.

In fact, as I suggested at the end of the previous section, the notion of causal relevance that I take Twardy and Korb ([2011], p. 906) to be seeking to analyse can be understood as a disjunction: $\vec{X}=\vec{x}$ is causally relevant to ϕ if and only if either $\vec{X}=\vec{x}$ is an actual cause of ϕ (a notion that I take to be captured by PC) or $\vec{X}=\vec{x}$ lowers the probability of ϕ (raises the probability of ¬ϕ) along a causal pathway representing a process that is complete except that ϕ occurs (despite $\vec{X}=\vec{x}$) (a notion that I take to essentially captured by the whole of PC, if we replace the ‘ >’s with ‘ <’s). So, in addition to incorporating into their analysis a condition that is similar to PC2(a), but which appeals to contingent probabilistic difference-making rather than contingent probability raising (that is, which makes use of ‘≠’s rather than ‘ >’s), Twardy and Korb also need a condition that captures the notion of a complete causal process from $\vec{X}=\vec{x}$ to ϕ. In Halpern and Pearl’s account, this complete causal process requirement is captured by AC2(b). My proposed generalization of AC2(b) to the probabilistic case is PC2(b). Twardy and Korb propose a different generalization of AC2(b) to the probabilistic case. They present two conditions to replace AC2(b).

As noted, the purpose of both AC2(b) and PC2(b) is to ensure that the causal process connecting the putative cause $\vec{X}=\vec{x}$ to the effect ϕ is complete. In the case of AC2(b), this is achieved by requiring that ϕ would hold (in circumstances $\vec{W}=\vec{w}\prime$) if $\vec{X}=\vec{x}$ held and any subset $\vec{Z}\prime$ of the variables $\vec{Z}$ representing the active causal process from $\vec{X}=\vec{x}$ to ϕ took their actual values $\vec{Z}\prime ={\vec{z}}^{*}$. In the case of PC2(b), it is achieved by requiring that if $\vec{X}=\vec{x}$ held and any subset $\vec{Z}\prime$ of the variables $\vec{Z}$ took their actual values $\vec{Z}\prime ={\vec{z}}^{*}$ (in circumstances $\vec{W}=\vec{w}\prime$), then the probability of ϕ would be higher than if $\vec{X}$ simply took the alternative value $\vec{X}=\vec{x}\prime$ (in circumstances $\vec{W}=\vec{w}\prime$).

The analogue to AC2(b) proposed by Twardy and Korb ([2011]) is markedly different. They do not appeal to what would happen, or what the probabilities would be, if any subset $\vec{Z}\prime$ of the variables $\vec{Z}$ representing the active causal process from $\vec{X}=\vec{x}$ to ϕ took their actual values $\vec{Z}\prime ={\vec{z}}^{*}$ (due to interventions or the like). Instead, they appeal to the notion of a ‘soft intervention’ (Twardy and Korb [2011], p. 907), where the latter (in contrast to the ‘hard’ interventions that can be taken to be represented by expressions of the form $do(\vec{X}=\vec{x})$) don’t fix the value of the variable intervened upon, but rather fix a probability distribution for the variable intervened upon. Their idea is that, rather than considering what would happen or what the probabilities would be, if subsets $\vec{Z}\prime$ of variables in $\vec{Z}$ took their actual values, $\vec{Z}\prime ={\vec{z}}^{*}$ (due to hard interventions), we should instead consider what the probabilities would be if subsets $\vec{Z}\prime$ of variables in $\vec{Z}$ took their original probability distributions (due to soft interventions) (Twardy and Korb [2011], p. 907).

Adapting the notation of Godszmidt and Pearl ([1992]) to the case of soft interventions, let $do(P(\vec{Z}\prime )=P(\vec{Z}\prime |do(\vec{X}=\vec{x})))$ represent a ‘soft’ intervention that sets the probability distribution over variables in $\vec{Z}\prime$ to that distribution that would obtain if the variables $\vec{X}$ were to take the values $\vec{X}=\vec{x}$ as a result of hard interventions (or local surgeries or small miracles). Then, some less important and some purely notational differences aside, the proposal made by Twardy and Korb ([2011], pp. 906–8) is that in the probabilistic context, Halpern and Pearl’s AC2 be replaced not by my PC2, but by the following:

PC2* There exists a partition $\left(\vec{Z},\vec{W}\right)$ of Y (that is, the set of endogenous variables in the model $M^{*}$) with $\vec{X}\subseteq \vec{Z}$ and some setting, $\left(\vec{x}\prime ,\vec{w}\prime \right)$, of the variables in $\left(\vec{X},\vec{W}\right)$ such that the following holds:
  1. $P(\phi |do(\vec{X}=\vec{x}\text{\&}\vec{W}=\vec{w}\prime ))\neq P(\phi |do(\vec{X}=\vec{x}\prime \text{\&}\vec{W}=\vec{w}\prime ))$. In words, if the variables in $\vec{W}$ had taken the values $\vec{W}=\vec{w}\prime$, then the probability of ϕ would be different if the variables in $\vec{X}$ took their actual values, $\vec{X}=\vec{x}$, than if the variables in $\vec{X}$ took the values $\vec{X}=\vec{x}\prime$.
  2. $P(\phi |do(\vec{X}=\vec{x}\text{\&}\vec{W}=\vec{w}))=P(\phi |do(\vec{X}=\vec{x}\text{\&}\vec{W}=\vec{w}\prime ))$, where $\vec{W}=\vec{w}$ are the actual values of $\vec{W}$. In words, if the variables in $\vec{X}$ had taken their actual values, $\vec{X}=\vec{x}$, then the probability of ϕ would have been no different if the variables in $\vec{W}$ had taken their actual values, $\vec{W}=\vec{w}$, than if they had taken the values $\vec{W}=\vec{w}\prime$.
  3. $P(\phi |do(\vec{X}=\vec{x}\text{\&}\vec{W}=\vec{w}\prime \text{\&}P(\vec{Z}\prime )=P(\vec{Z}\prime |do(\vec{X}=\vec{x})))=P(\phi |do(\vec{X}=\vec{x}\text{\&}\vec{W}=\vec{w}\prime ))$ for all subsets $\vec{Z}\prime$ of $\vec{Z}/\{\vec{X},\phi \}$. In words, if the variables in $\vec{X}$ had taken their actual values, $\vec{X}=\vec{x}$, and the variables in $\vec{W}$ had taken the values $\vec{W}=\vec{w}\prime$, then the probability of ϕ would be no different if, additionally, the probability distribution over any arbitrary subset of the variables in $\vec{Z}$ (excluding those in $\vec{X}$ or ϕ) had (due to a soft intervention) been the same as it would be if merely $\vec{X}=\vec{x}$.

Since Twardy and Korb ([2011], p. 902) only make provision for primitive events to act as cause and effect (thus effectively requiring that $\vec{X}=\vec{x}$ and ϕ stand for primitive events, rather than potentially standing, respectively, for conjunctions or for Boolean combinations of primitive events), they don’t need a minimality condition analogous to Halpern and Pearl’s AC3 or my PC3. They do, however, incorporate the requirement that both *X* = *x* and *Y* = *y* be actual if *X* = *x* is to count as causally relevant to *Y* = *y* in the sense that they wish to analyse (Twardy and Korb [2011], p. 902). Consequently, they effectively replicate condition PC1. Thus, if we limit our attention to causation between primitive events, it is PC2${}^{*}$ (most significantly, PC2${}^{*}$(b) and PC2${}^{*}$(c)) that differentiates Twardy and Korb’s account from my own.

Twardy and Korb’s account yields the correct verdicts concerning the probabilistic preemption case described in Section 6 above. Specifically, PC2${}^{*}$(a) is satisfied when we let $\vec{X}=\{C\},\;\vec{x}=\{C=1\}$, and when we let ϕ be *D* = 1. For let $\vec{W}=\langle B,T\rangle ,\;\vec{w}\prime =\{B=1,T=0\}$, and $\vec{Z}=\langle C,S,D\rangle$. Condition PC2${}^{*}$(a) is satisfied because if *B* = 1 and *T* = 0 and *C* = 1, then the probability of *D* = 1 would have been approximately 0.45, whereas if *B* = 1 and *T* = 0 and *C* = 0, then the probability of *D* = 1 would have been approximately 0.^59^ Condition PC2${}^{*}$(b) is trivially satisfied, since $\vec{w}\prime =\{B=1,T=0\}$ are the actual values of $\vec{W}=\langle B,T\rangle$. Finally, PC2${}^{*}$(c) is satisfied because interventions on the values of *B* and *T* do not make a difference to the probability of *S*. This means that if *C* = 1 and *B* = 1 and *T* = 0, a soft intervention setting the probability that *S* = 1 to the value that it would have had if merely *C* = 1 (and $\vec{W}=\langle B,T\rangle$ had not been forced to take $\vec{w}\prime =\{B=1,T=0\}$ by hard interventions) in fact makes no difference to the probability of *S* = 1 at all (it remains at 0.9). Consequently (when *C* = 1 and *B* = 1 and *T* = 0), setting the probability that *S* = 1 to this value makes no difference to the probability that *D* = 1 (which remains approximately 0.9 × 0.5 = 0.45). So PC2${}^{*}$(c), in addition to PC2${}^{*}$(a) and PC2${}^{*}$(b), is satisfied when we consider *C* = 1 as a potential cause of *D* = 1. Since it is also the case that *C* = 1 and *D* = 1 are the actual values of *C* and *D* (in the world in which this causal scenario plays out), Twardy and Korb’s account yields the correct result that *C* = 1 is a cause of *D* = 1.

It also yields the correct result that *B* = 1 is not a cause of *D* = 1. To see this, observe the following: Condition PC2${}^{*}$(a) is satisfied when we let $\vec{X}=\{B\},\;\vec{x}=\{B=1\}$, and we let ϕ be *D* = 1. For let $\vec{W}=\langle C,S\rangle ,\;\vec{w}\prime =\{C=1,S=0\}$, and $\vec{Z}=\langle B,T,D\rangle$. If *C* = 1 and *S* = 0 and *B* = 1, then the probability of *D* = 1 would have been approximately 0.81; but if *C* = 1 and *S* = 0 and *B* = 0, then the probability of *D* = 1 would have been approximately 0. So PC2${}^{*}$(a) is satisfied. However, PC2${}^{*}$(b) is violated. After all, if *B* = 1 and the variables $\vec{W}=\langle C,S\rangle$ had taken their actual values, $\vec{w}=\{C=1,S=1\}$, then the probability of *D* = 1 would have been approximately 0.5, which is different from the probability that *D* = 1 if *B* = 1, *C* = 1, and *S* = 0 (which is approximately 0.81).

Could we instead let $\vec{w}\prime$ be the actual values of $\vec{W}=\langle C,S\rangle$, that is, let $\vec{w}\prime =\{C=1,S=1\}$? Perhaps we could argue that PC2${}^{*}$(a) is still satisfied: that if *C* = 1 and *S* = 1 and *B* = 1, then the probability of *D* = 1 would have been different than if *C* = 1 and *S* = 1 and *B* = 0. This will be so if in the case where Barzini issues his order and Sonny shoots, there’s still some (albeit small) chance of Turk shooting too (and if it’s the case that if they both shoot, then the probability of McCluskey’s death is different than if Sonny shoots alone). This chance—the chance that Turk would also shoot if Sonny shot and Barzini issued his order—is of course lower than the chance that Turk would shoot if Barzini issued his order (and no intervention on whether Sonny shoots occurrs), which is approximately 0.09 (remember, Corleone’s order is implicitly held fixed by a suitable semantics for this counterfactual). After all, in the example Sonny’s shooting lowers the probability of Turk’s shooting.

Condition PC2${}^{*}$(b) is now trivially satisfied, since $\vec{w}\prime =\{C=1,S=1\}$ are the actual values of $\vec{W}=\langle C,S\rangle$. But PC2${}^{*}$(c) is now violated for if *B* = 1, *C* = 1, and *S* = 1, then if *T* = 1 were due to a soft intervention to take the value it would have received if simply *B* took *B* = 1 due to a (hard) intervention (and the values of *C* and *S* were not intervened upon)—namely, approximately 0.09—then the probability of *D* = 1 would be different (higher) than it would be if merely (due to hard interventions) *B* = 1, *C* = 1, and *S* = 1 (and the probability of *T* = 1 took the lower value—close to 0—that it would receive without this soft intervention).

So it seems that where we consider *B* = 1 as a potential cause of *D* = 1, either PC2${}^{*}$(b) or PC2${}^{*}$(c) is violated (depending on how we assign values to $\vec{W}$). So Twardy and Korb’s analysis correctly diagnoses *B* = 1 as a non-cause of *D* = 1.

In the next section, I will describe two examples that my account, PC, can handle, the first of which shows that Twardy and Korb’s account doesn’t provide a sufficient condition for actual causation, the second of which shows that it doesn’t provide a necessary condition. Since they advance their account as an analysis of causal relevance rather than actual causation, these needn’t be taken to show that Twardy and Korb’s account doesn’t succeed as an analysis of its own target notion. However, the examples do show that their account as it stands can’t be taken to provide an adequate analysis of actual causation. They also serve to further illustrate the virtues of the analysis of actual causation developed here, which correctly handles the examples.

It should, however, be noted that although Twardy and Korb don’t make fully explicit the relationship between actual causation and the notion of causal relevance that they seek to analyse, it does in fact appear (as I have noted) that they take actual causation to be a special case of causal relevance (Twardy and Korb [2011], pp. 902, 906), with the other case being that in which the causally relevant factor, $\vec{X}=\vec{x}$, lowers the probability of the factor, ϕ, that it is causally relevant to (thus raising the probability of ¬ϕ) along a causal pathway representing a process that is complete (except that ϕ holds rather than ¬ϕ). Importantly, both cases require a causal process from $\vec{X}=\vec{x}$ to ϕ that is complete (except that, in the second case, the obtaining of ϕ itself might be taken to constitute an incompleteness). On Twardy and Korb’s account, it is PC2${}^{*}$(b) and PC2${}^{*}$(c) that are intended to capture the requirement that the causal process be complete. On my account, by contrast, PC2(b) plays the role of ensuring a complete causal process from $\vec{X}=\vec{x}$ to ϕ. But the examples that I give in the next section show precisely that the conjunction of PC2${}^{*}$(b) and PC2${}^{*}$(c) is not necessary or sufficient to capture the requirement that a causal process be complete, whereas PC2(b) is necessary and sufficient. (It is thus worth noting that the examples that I will present do not trade on the difference between my PC2(a) and Twardy and Korb’s PC2${}^{*}$(a). That is, they do not trade upon the fact that my account appeals to contingent probability raising, whereas theirs appeals to contingent probabilistic difference-making.) So in fact I do think that the examples that I shall present are counterexamples to the analysis of Twardy and Korb, even when that analysis is taken on its own terms, as an analysis of a more inclusive notion than that of actual causation.

## 10 Probabilistic Fizzling

In our probabilistic preemption case, the reason that the ‘backup’ process initiated by Barzini’s order didn’t run to completion (in that Turk did not shoot McCluskey) can be explained in terms of the fact that Sonny shot before Turk arrived at the scene, thus greatly reducing the chance of Turk’s shooting McCluskey (a case of probabilistic prevention). This is strongly analogous to the deterministic preemption case in which Sonny’s shooting deterministically prevents Turk from shooting.

However, probabilistic processes (such as that initiated by Barzini’s order in the probabilistic version of our preemption scenario) do not need to be ‘interrupted’ by other processes (such as that initiated by Corleone’s order) in order for them to fail to run to completion. Because such processes are probabilistic, they may—to adopt the terminology of Schaffer ([2001], p. 91)—simply ‘fizzle out’ as a matter of probability.

Consider a modified version of our probabilistic preemption example that is exactly as before (in that all of the probabilities are the same, and both Barzini and Corleone issue their orders) except that, as a matter of chance, Sonny doesn’t shoot (recall that in the original probabilistic example, there was a 0.1 chance of his not shooting, given Corleone’s order). Suppose that in spite of Sonny’s not shooting, and again as a matter of chance, Turk doesn’t shoot either (there was a 0.1 chance of Turk’s not shooting given Barzini’s order and Sonny’s not shooting). Finally, as a matter of (very small) chance, McCluskey dies anyway (of an unrelated heart attack).^60^

In this case, both the process initiated by Corleone’s order and the process initiated by Barzini’s order, simply ‘fizzle out’ as a matter of probability before they can run to completion and cause McCluskey’s death. To use Schaffer’s terminology again, we can regard Turk’s failure to shoot as the ‘fizzling’ event (Schaffer [2001], p. 81) or (for short) ‘fizzler’ (Schaffer [2001], p. 81) on the Barzini process, and Sonny’s failure to shoot as the ‘fizzler’ on the Corleone-process.

Intuitively, in this revised scenario neither Corleone’s nor Barzini’s order was an actual cause of McCluskey’s death. Yet, just as before, both bear the contingent probability-raising relations to it required by PC2(a). Specifically, the relevant Inequalities (8) and (10) (see Section 8, above) continue to obtain.

Still, PC correctly diagnoses both Corleone’s order and Barzini’s order as non-causes. This is because PC2(b) is violated in each case. In the case of Barzini’s order, it is violated for exactly the same reason as before, namely, because Inequality (11) (see Section 8, above) continues to hold in this version of the example, with the (‘fizzling’) value *T* = 0 (representing Turk’s non-shooting) still being the actual value of *T*.

But in this case PC2(b) is also violated when we consider Corleone’s order as a putative actual cause of McCluskey’s death. For let $\vec{W}=\langle B,T\rangle ,\;\vec{w}\prime =\{B=1,T=0\},\;\vec{Z}=\langle C,S,D\rangle$, and $\vec{Z}\prime =\{S\}\subset \vec{Z}$, and note that the following inequality holds:

(12) P(D=1|do(C=1&B=1&T=0&S=0)) ≤P(D=1|do(C=0&B=1&T=0)).

That is, if *B* = 1 and *T* = 0 and *C* = 1 and *S* took its actual value, which is now *S* = 0, then the probability of *D* = 1 would have been no higher than it would have been if *B* = 1 and *T* = 0 and *C* = 0. Or, in other words, where Barzini issues his order but Turk doesn’t shoot, the probability of McCluskey’s dying if Corleone issues his order but Sonny doesn’t shoot is no higher than it would have been if Corleone hadn’t issued his order in the first place. Since PC2(b) is violated in this variant of the example when we consider *C* = 1 as a putative actual cause of *D* = 1, PC, correctly, does not count *C* = 1 as an actual cause of *D* = 1 in this case.

By contrast, though Twardy and Korb’s account counts Barzini’s order as causally irrelevant to McCluskey’s dying in this case, it counts Corleone’s order as causally relevant to McCluskey’s dying. To see that it counts Barzini’s order as causally irrelevant, let $\vec{X}=\{B\},\;\vec{x}=\{B=1\}$, and let ϕ be *D* = 1. Let $\vec{W}=\langle C,S\rangle ,\;\vec{w}\prime =\{C=1,S=0\}$, and $\vec{Z}=\langle B,T,D\rangle$. Condition PC2${}^{*}$(a) is satisfied because Inequality (10) from Section 8 above continues to hold in this version of the example. Condition PC2${}^{*}$(b) is satisfied trivially, because {C=1,S=0} are the actual values of *C* and *S* in this version of the example. But PC2${}^{*}$(c) is violated for if *B* = 1, *C* = 1, and *S* = 0, and if the probability of *T* = 1 were, due to a soft intervention, to take the value that it would have received if simply *B* took *B* = 1 due to a (hard) intervention (and the values of *C* and *S* were not intervened upon)—namely, approximately 0.09—then the probability of *D* = 1 would have been approximately 0.081. This is different than the probability for *D* = 1 that would have obtained if (due to hard interventions) *B* = 1, *C* = 1, and *S* = 0 (and there were no soft intervention on the probability of *T* = 1), which would have been approximately 0.81.

Could we instead let $\vec{w}\prime$ be $\vec{w}\prime =\{C=1,S=1\}$? Perhaps we could argue that PC2${}^{*}$(a) is still satisfied if we do so. That is, we could perhaps argue that if *C* = 1 and *S* = 1 and *B* = 1, then the probability of *D* = 1 would have been different than if *C* = 1 and *S* = 1 and *B* = 0. This will be so if, in the case where Barzini issues his order and Sonny shoots, there’s still some (albeit small) chance of Turk shooting too (and if it’s the case that if they both shoot, then the probability of McCluskey’s death is different than if Sonny shoots alone). The trouble is that PC2${}^{*}$(b) is now violated. After all, if *B* = 1 and the variables $\vec{W}=\langle C,S\rangle$ had taken the values that they actually have (in the version of the example presently under consideration), $\vec{w}=\{C=1,S=0\}$, then the probability of *D* = 1 would have been approximately 0.81, which is different from the probability that *D* = 1 would have had if *B* = 1, *C* = 1, and *S* = 1, which is approximately 0.5.

So it seems that where we consider *B* = 1 as a potential cause of *D* = 1, either PC2${}^{*}$(b) or PC2${}^{*}$(c) is violated (depending on how we assign values to $\vec{W}=\langle C,S\rangle$). Twardy and Korb’s analysis (correctly) diagnoses *B* = 1 as causally irrelevant to *D* = 1 in this case.

To see that Twardy and Korb’s analysis (incorrectly) diagnoses *C* = 1 as causally relevant to *D* = 1 in this case, note that PC2${}^{*}$(a) is satisfied when we let $\vec{X}=\{C\},\;\vec{x}=\{C=1\}$, and when we let ϕ be *D* = 1. For let $\vec{W}=\langle B,T\rangle ,\;\vec{w}\prime =\{B=1,T=0\}$, and $\vec{Z}=\langle C,S,D\rangle$. Then condition PC2${}^{*}$(a) is satisfied in virtue of the fact that Inequality (8) (from Section 8, above) continues to hold. Condition PC2${}^{*}$(b) is trivially satisfied, since $\vec{w}\prime =\{B=1,T=0\}$ are the actual values of $\vec{W}=\langle B,T\rangle$. Finally, PC2${}^{*}$(c) is satisfied because the values of *B* and *T* are (when set by interventions) probabilistically irrelevant to that of *S*. This means that if *C* takes its actual value, *C* = 1, while $\vec{W}=\langle B,T\rangle$ takes (due to interventions) the values $\vec{w}\prime =\{B=1,T=0\}$, then a soft intervention changing the probability that *S* = 1 back to the value that it would have if *C* took *C* = 1 (without the additional assumption that, due to interventions, $\vec{W}=\langle B,T\rangle$ took $\vec{w}\prime =\{B=1,T=0\}$) doesn’t, in fact, change the probability of *S* = 1 at all (it remains at 0.9 either way). Consequently, given *C* = 1, *B* = 1, and *T* = 0, whether or not this soft intervention occurs makes no difference to the probability of *D* = 1 (either way, it is approximately 0.9 × 0.5 = 0.45). Condition PC2${}^{*}$(c) is thus satisfied. So condition PC2${}^{*}$ is satisfied. And, since *C* = 1 and *D* = 1 are the actual values of *C* and *D* in this version of the example, Twardy and Korb’s account thus yields the result that *C* = 1 is causally relevant to *D* = 1 in this case.

Since, as I read them, Twardy and Korb take causal relevance involving contingent probability raising, as opposed to contingent probability lowering, to imply actual causation (that is, they take the satisfaction of PC2${}^{*}$(b) and PC2${}^{*}$(c) together with the satisfaction of the condition that results from substituting ≠ with > rather than with < in PC2${}^{*}$(a) to be sufficient for actual causation), this result appears to be one that is incorrect by their lights.^61^ More importantly for my purposes, it also shows that replacing my condition PC2(b) with their conditions PC2${}^{*}$(b) and PC2${}^{*}$(c) in the analysis PC would result in a set of conditions that was no longer sufficient for actual causation.

The reasoning that shows that Twardy and Korb’s account (incorrectly) counts *C* = 1 as causally relevant to *D* = 1 in the most recent fizzling example is exactly the same as the reasoning that shows that it (correctly) counts *C* = 1 as causally relevant to *D* = 1 in the original probabilistic preemption scenario. This shows that Twardy and Korb’s account, unlike the account proposed here, isn’t sufficiently sensitive to whether putative cause and effect are connected by a complete causal process to ensure that non-causes are always correctly diagnosed as such.

The example just considered shows that Twardy and Korb’s account (unlike PC) doesn’t constitute a sufficient condition for actual causation. A further variant on our probabilistic preemption scenario shows that it doesn’t constitute a necessary condition either. Suppose this time that things are exactly as before (in that all of the probabilities are the same as in the original probabilistic preemption scenario, and both Barzini and Corleone issue their orders) and that (as in the ‘fizzling’ example described at the beginning of this section), as a matter of chance, Sonny doesn’t shoot (*S* = 0). But suppose that this time, and again as a matter of chance, Turk does shoot (*T* = 1) and Turk’s bullet hits and kills McCluskey.

My proposed definition, PC, yields the correct results about this latest case. *C* = 1 is correctly counted as a non-cause of *D* = 1. To see this, let $\vec{W}=\langle B,T\rangle ,\;\vec{w}\prime =\{B=1,T=0\},\;\vec{Z}=\langle C,S,D\rangle$, and $\vec{Z}\prime =\{S\}\subset \vec{Z}$. Condition PC2(a) is satisfied because Inequality (8) (from Section 8, above) still holds in this latest version of the example. But condition PC2(b) is violated because Inequality (12) (this section, above) holds, and *S* = 0 is the actual value of *S* in this case.

On the other hand, my proposed definition, PC, correctly counts *B* = 1 as an actual cause of *D* = 1 in this case. To see this, let $\vec{W}=\langle C,S\rangle ,\;\vec{w}\prime =\{C=1,S=0\}$, and $\vec{Z}=\langle B,T,D\rangle$. Condition PC2(a) is satisfied because Inequality (10) (Section 8, above) holds. Condition PC2(b) is also satisfied because the probability of *D* = 1 is higher when *B* = 1, *C* = 1, *S* = 0, and arbitrary subsets of $\vec{Z}=\langle B,T,D\rangle$ take their actual values, than it is when *B* = 0, *C* = 1, and *S* = 0. In particular consider $\vec{Z}\prime =\{T\}\subset \vec{Z}$. The actual value of *T* in this version of the scenario is *T* = 1, and note that:

(13) P(D=1|do(B=1&C=1&S=0&T=1))>P(D=1|do(B=0&C=1&S=0)).

The term on the left-hand side of this inequality is approximately equal to 0.9, while the term on the right-hand side is approximately equal to 0. Clearly, we could remove *T* = 1 and/or add *D* = 1 and/or (another iteration of) *B* = 1 within the scope of the do(·) operator in the probability expression that appears on the left-hand side of this inequality without affecting the fact that the inequality holds. It thus holds when we include the actual values of arbitrary subsets of $\vec{Z}$ within the scope of the do(·) operator on the left-hand side, as PC2(b) requires. So PC2(b) holds and, consequently, PC correctly diagnoses *B* = 1 as an actual cause of *D* = 1 in this version of the scenario.

Twardy and Korb’s account, by contrast, classifies *B* = 1 as not causally relevant to *D* = 1. To see this, note that PC2${}^{*}$(a) is satisfied when we let $\vec{X}=\{B\},\;\vec{x}=\{B=1\}$, and when ϕ is *D* = 1. For let $\vec{W}=\langle C,S\rangle ,\;\vec{w}\prime =\{C=1,S=0\}$, and let $\vec{Z}=\langle B,T,D\rangle$. Then PC2${}^{*}$(a) is satisfied because Inequality (10) (from Section 8, above) continues to hold in this variant of the example. Condition PC2${}^{*}$(b) is also trivially satisfied, since in this variant of the example $\vec{w}\prime =\{C=1,S=0\}$ are the actual values of $\vec{W}=\langle C,S\rangle$. But PC2${}^{*}$(c) is violated for if *B* = 1, *C* = 1, and *S* = 0, then the probability of *T* = 1 is 0.9, and the probability of *D* = 1 is approximately 0.81. But if *B* = 1, *C* = 1, and *S* = 0, and the probability of *T* = 1 were (due to a soft intervention) to take the value that it receives if we simply set *B* = 1 and perform no further interventions, which is approximately 0.09, then the probability that *D* = 1 would be significantly lower (approximately 0.081). So, where we consider *B* = 1 as potentially causally relevant to *D* = 1, PC2${}^{*}$(c) is violated. Twardy and Korb’s analysis classifies *B* = 1 as not causally relevant to *D* = 1 in this scenario. I take it that this classification is incorrect, since I take it that the fact that *B* = 1 is an actual cause of *D* = 1 (which it intuitively is in this case) is sufficient for *B* = 1 to count as causally relevant to *D* = 1.

The reasoning that shows that Twardy and Korb’s account (incorrectly) counts *B* = 1 as causally irrelevant to *D* = 1 in the most recent example (in which, actually, *T* = 1) is exactly the same as the reasoning that shows that it (correctly) counts *B* = 1 as causally irrelevant to *D* = 1 in the previous example (in which, actually, *T* = 0). The reason that Twardy and Korb’s account goes wrong is, once again, that unlike my account, their account tests what the probability of the putative effect would be not if the variables on the active causal process took their actual values (while $\vec{X}$ takes $\vec{X}=\vec{x}$ and $\vec{W}$ takes $\vec{W}=\vec{w}\prime$), but if these variables took their actual probability distributions (while $\vec{X}$ takes $\vec{X}=\vec{x}$ and $\vec{W}$ takes $\vec{W}=\vec{w}\prime$). This means that their account isn’t sufficiently sensitive to whether putative cause and effect are connected by a complete causal process.^62^

In fairness to Twardy and Korb, they do claim (Twardy and Korb [2011], pp. 900, 912) that a complete account of actual causation will require the structural equations/probabilistic causal models framework to be supplemented with an account of the metaphysics of causal processes (see also Handfield *et al.* [2008]). However, in (Twardy and Korb [2011]), their stated aim is to ‘push stochastic causal models as far as they can go alone’ (Twardy and Korb [2011], p. 900). My claim is that the analysis suggested here pushes them further than does Twardy and Korb’s analysis and, in doing so, better captures, within a probabilistic causal modelling framework, the intuition that cause and effect must be linked by a complete causal process.

## 11 Conclusion

It has been shown that Halpern and Pearl’s definition of actual cause admits of a natural extension to the probabilistic case. The probabilistic rendering that I have proposed elegantly handles cases of probabilistic preemption, as well as cases of fizzling. The latter cases are incorrectly diagnosed by the account of Twardy and Korb ([2011]), which in other respects is the probabilistic account of causation that is most similar to that proposed here. Though a survey of how my account handles the full battery of problem cases against which analyses of actual causation are tested is beyond the scope of this article, the fact that Halpern and Pearl have shown that their analysis of deterministic actual causation is able to handle a large range of deterministic cases lends at least some plausibility to the conjecture that the probabilistic analogue of their definition developed here may have success in handling the probabilistic variants of such cases. Further credence is lent to this conjecture by the fact that Twardy and Korb ([2011], [unpublished]) have shown that their account, which bears similarities to mine (except in its handling of fizzling), is able to handle a number of such cases.

In addition to applying the analysis developed here to a greater range of test cases, it will also be worth exploring whether the refinement added to Halpern and Pearl’s account in later articles by Halpern ([2008]) and Halpern and Hitchcock ([2010], [2015])—namely, the incorporation of normality considerations—which is designed to enable the account to handle a still greater range of problem cases, can and should be adapted to this proposed probabilistic extension of the analysis. I look forward to pursuing both of these lines of investigation in future work.

## Footnotes

^1^ There is a minor difference between the analysis offered in (Halpern and Pearl [2001], pp. 196–7) and that offered in (Halpern and Pearl [2005], p. 853), which I will return to in Section 4 below. In the meantime, I shall talk as though Halpern and Pearl ([2001], [2005]) offer just a single analysis.

^2^ The analysis given in (Pearl [2009], Chapter 10) was first published in (Pearl [2000], Chapter 10). Pearl ([2009], pp. 329–30) takes the analysis of Halpern and Pearl ([2001], [2005]) to be a refinement of, and improvement upon, that published in (Pearl [2000], [2009]); he points out that the analysis of Halpern and Pearl ([2001], [2005]) handles cases that are counterexamples to the analysis of Pearl ([2000], [2009]).

^3^ The refined account, like Halpern and Pearl’s original analysis, incorporates the assumption of determinism (Halpern and Hitchcock [2015], Section 2).

^4^ Blanchard and Schaffer ([forthcoming]) additionally argue that the refinement is problematic, and in any case doesn’t achieve its desired upshot.

^5^ My exposition of SEMs differs a little from that given by Halpern and Pearl ([2005], pp. 846–52); in the interests of simplicity, I omit technical details that are inessential for the purposes of this paper.

^6^ In the vector notation used by Halpern and Pearl ([2005], pp. 848–9, 852), $\vec{V}$ denotes the ordered sequence of variables $\langle V_{1},V_{2},\ldots ,V_{n}\rangle$, while $\vec{v}$ denotes an ordered sequence of values $\langle v_{1},v_{2},\ldots ,v_{n}\rangle$ such that for all *i*, *v_i_* is a possible value of the variable *V_i_*. The assignment of values $V_{1}=v_{1},V_{2}=v_{2},\ldots ,V_{n}=v_{n}$ can be abbreviated as $\langle V_{1},V_{2},\ldots ,V_{n}\rangle =\langle v_{1},v_{2},\ldots ,v_{n}\rangle$, or (even more concisely) $\vec{V}=\vec{v}$.

^7^ On this point, see (Pearl [1995], pp. 673–4, [2009], pp. 32, 37, 69–70, 204–5, 317, 416–18; Hitchcock [2001a], p. 283; Halpern and Pearl [2005], pp. 848–9; Woodward [2005], p. 48; Halpern and Hitchcock [2015], p. 419).

^8^ Thanks to an anonymous referee for encouraging me to say more about this.

^9^ I describe this semantics as ‘broadly Lewisian’ because Lewis ([1979]) himself focuses upon counterfactuals concerning events, rather than variable-values, and (for the most part) upon counterfactuals with relatively simple antecedents requiring only a single small miracle to implement. Glynn ([2013], pp. 49–51) has argued that Lewis’s semantics can be extended—in roughly the way described in the main text above—to the sorts of counterfactuals that SEMs can be taken to encode.

^10^ Woodward ([2005], p. 98) gives a technically rigorous definition of the notion of an intervention, as do Pearl ([1995], pp. 673, 670, [2009], pp. 68–78, 88–9) and Spirtes *et al.* ([2000], pp. 47–53). For a comparison of these various formal characterizations, see (Woodward [2005], pp. 107–11). For the time being, it will suffice to think of an intervention as a causal process that is exogenous to the system being modelled, and which interferes with its usual workings, so that the value of the variable intervened upon is altered without any alteration to those variables in the model that are its parents (or, more generally, its non-descendants) (cf. Woodward [2005], p. 47).

^11^ See also (Pearl [1995], p. 672; Halpern and Pearl [2005], pp. 841, 851, 878).

^12^ See (Woodward [2005], pp. 55, 110) for a detailed exposition of the difference between Pearl’s approach and Woodward’s.

^13^ Cf. (Pearl [2009], pp. 420–1, [1995], p. 677).

^14^ For further discussion of the non-reductivity of Woodward’s approach and of whether it is compatible with an illuminating account of actual causation, see (Strevens [2007], pp. 245–6, [2008], pp. 180–2; Woodward [2008], pp. 203–4; Glynn [2013], pp. 47–8).

^15^ I adopt slightly different notation than Halpern and Pearl ([2005]; cf. Pearl [2009], p. 330) in that I use ‘=’ rather than ‘←’ as an assignment operator in writing out causal formulas. Halpern and Pearl ([2005], p. 852) write (for example) $\vec{Y}\leftarrow \vec{y}$ rather than $\vec{Y}=\vec{y}$ in their basic causal formulas as a reminder that the formula says what would happen if the variables $\vec{Y}$ were set to the values $\vec{y}$ by interventions (alternatively, small miracles/local surgeries), rather than what would happen if the variables $\vec{Y}$ came to have the values $\vec{y}$ as a result of different initial conditions operating via the ordinary structural equations. As such, the ‘←’ notation serves the same function as Pearl’s ‘do(·)’ operator (as in $do(\vec{Y}=\vec{y})$) (Godszmidt and Pearl [1992], pp. 669–71; Pearl [2009], p. 70). Using ‘=’ rather than ‘←’ is harmless (and avoids the multiplication of notation) provided that it is borne in mind that a basic causal formula expresses a counterfactual that is to be given a non-backtracking semantics.

^16^ I have made minor notational adjustments to Halpern and Pearl’s definition for consistency with my own notation. I have also inserted some clarificatory text within double square brackets—that is, [[…]]. I use double square brackets for this purpose so as to avoid confusion with Halpern and Pearl’s use of single square brackets—that is, […]. They use single square brackets to delimit the antecedents of causal formulas.

^17^ Halpern and Pearl ([2005], p. 852) regard this definition as merely ‘preliminary’, for reasons that I discuss at the end of Section 5, below.

^18^ I here state the version of the definition given in (Halpern and Pearl [2001], pp. 196–7), with minor notational changes, rather than the version given in (Halpern and Pearl [2005], p. 853). The two versions differ only in condition AC2(b). In the later article, AC2(b) is slightly more complicated. The additional complication is intended to address a putative counterexample, given by Hopkins and Pearl ([2003], pp. 85–6), to the earlier version of the definition (see Halpern and Pearl [2005], p. 882). However, it is not clear that this additional complication is really necessary. Christopher Hitchcock, in personal communication with Brad Weslake (Weslake [forthcoming], Footnote 15), has suggested (to my mind very plausibly) that the example given by Hopkins and Pearl is really just a preemption case, of the sort that the original Halpern and Pearl ([2001], pp. 196–7) definition can handle without modification. Weslake ([forthcoming], Footnote 15) concurs that the modification proposed in (Halpern and Pearl [2005]) is not the correct way to respond to the example given by Hopkins and Pearl, and that treating it as a preemption case is a ‘better way to handle’ it (Weslake [forthcoming], Footnote 15) (though Weslake ([forthcoming], Section 4) himself ultimately endorses an account of actual causation that differs from both versions of Halpern and Pearl’s). Halpern ([unpublished], Section 4) also suggests that the modification introduced by Halpern and Pearl ([2005]) is not necessary. So it appears that there is reason to prefer the original, simpler definition presented in (Halpern and Pearl [2001]). What is interesting is that the probabilistic analogue of the original AC2(b) that I shall describe below better handles probabilistic preemption cases than does the natural probabilistic analogue of the later version of AC2(b). Insofar as we are interested in developing a uniform treatment of deterministic and probabilistic causation, this might be taken as an additional reason to prefer the earlier version of AC2(b).

^19^ Oddly, despite the fact that Halpern and Pearl ([2001], [2005]) consistently describe this as a ‘definition’, it (both in the variant presented in (Halpern and Pearl [2001]) and the variant presented in (Halpern and Pearl [2005])) takes the form of merely a sufficient condition. Nevertheless, I take it that it is charitable to regard Halpern and Pearl as intending to offer it as a necessary and sufficient condition, since their discussion of how this ‘definition’ handles the standard battery of test cases (Halpern and Pearl [2001], pp. 197–202; Halpern and Pearl [2005], pp. 859–69) only really makes sense on this assumption.

^20^ It is worth emphasizing that this is merely an informal gloss. Nothing in Halpern and Pearl’s definition requires that we go beyond a counterfactual understanding of actual cause and posit the existence of anything like irreducible causal processes. As Halpern and Pearl define it, this notion of an active causal process is just a generalization of Hitchcock’s notion of an ‘active route’ (Hitchcock [2001a], p. 286), which can be defined in purely counterfactual terms (Hitchcock [2001a], p. 286).

^21^ I’m making the point here with respect to the sort of Boolean combinations of primitive events that Halpern and Pearl themselves take to be potential causes and effects.

^22^ As Blanchard and Schaffer ([forthcoming], Section 1.3) point out, this requirement commits Hitchcock (on pain of triviality) to the existence of a semantics for the counterfactuals ‘entailed’ by M that doesn’t simply appeal to the results of equation replacement with respect to M.

^23^ The reason for adding these two variables is that AC doesn’t allow the values of exogenous variables to act as actual causes. Since we’re interested in evaluating whether *C* = 1 and *B* = 1 are actual causes of *D* = 1, we therefore have to ensure the endogeneity of *C* and *B* by including in our model variables upon which the values of *C* and *B* depend. Halpern and Pearl’s disallowance of the values of exogenous variables from counting as actual causes is somewhat arbitrary, though harmless. It is harmless because, in general, we can ensure the endogeneity of a variable that we wish to evaluate as a putative cause by including in our variable set variables upon which its value depends, as I have done here for the variables *C* and *B*.

^24^ Note that in this case, the assignment appealed to—namely, $\vec{w}\prime =\{C=1,S=0\}$—involves *S* taking the non-actual value *S* = 0. Definition AC allows us to consider non-actual assignments $\vec{w}\prime$ of values to the variables $\vec{W}$. Without doing so, it would be unable to handle cases of symmetric overdetermination (Halpern and Pearl [2005], pp. 856–8). The probabilistic extension of AC that I will suggest below also allows us to consider such non-actual assignments. Though I shall not attempt to demonstrate it here, consideration of non-actual assignments is needed in the probabilistic case in order to correctly diagnose cases in which two causes symmetrically overdetermine the probability of an effect (an example of this is described in (Glynn [2009], Section 4.5.4.C).

^25^ For reasons outlined in Footnote 24 above, AC correctly allows us to suppose that Sonny doesn’t shoot (even though actually Sonny does shoot) in looking for contingent counterfactual dependence of McCluskey’s death upon Barzini’s order.

^26^ My notation differs slightly from Halpern and Pearl’s.

^27^ Interestingly, Schaffer ([2013], p. 48) suggests that construing causation as contrastive in nature may make appeals to ‘defaults’ or ‘normality’—of the sort discussed in the main text in the three paragraphs preceding this one—unnecessary in the analysis of actual cause.

^28^ Though ambiguity is avoided if context picks out the relevant contrast sets (see, for example, Schaffer [2005], p. 329).

^29^ From now on, wherever it is specified that $\vec{W}=Ø$, I shall leave this parenthetical qualification implicit.

^30^ We might also require that for every ϕ′ in ϕ′, there is some event of the form $\vec{X}=\vec{x}\prime$ in $\vec{X}=\vec{x}\prime$ such that AC2(a) holds when ¬ϕ is replaced with ϕ′, and the non-actual setting of $\vec{X}$ appealed to in AC2(a) is taken to be precisely the setting $\vec{X}=\vec{x}\prime$ (cf. Schaffer [2005], p. 348).

^31^ See, for example, (Good [1961a], [1961b]; Reichenbach [1971], p. 204; Suppes [1970], pp. 12, 21, 24; Lewis [1986a], pp. 175–84; Menzies [1989]; Eells [1991], Chapter 6; Kvart [2004]).

^32^ Existing accounts of probabilistic actual causation—including those mentioned in Footnote 31—are problematic, for reasons documented in (Salmon [1984], pp. 192–202; Menzies, [1996], pp. 85–96; Hitchcock [2004]; Glynn [2011], pp. 377–86). I shall not recount those reasons here; the interested reader is referred to the cited works. There is one recent account of probabilistic causation—namely, that developed by Twardy and Korb ([2011])—which I will discuss in some detail in Section 9 and Section 10. This, of all existing accounts, is the most similar in spirit to the account that I shall develop below. In Section 10, I will outline two counterexamples to it, which my own account avoids.

^33^ As Pearl ([2009], p. 70) notes, $do(\vec{V}=\vec{v})$ is equivalent to $set(\vec{V}=\vec{v})$, the latter being notation introduced by Pearl ([1995], pp. 673–4). Pearl ([2009], pp. 70, 127, 334) points out that there are many alternative notations used in statistics and elsewhere to denote much the same thing.

^34^ I shall leave this parenthetical qualification implicit from now on.

^35^ Since objective chances—which are the sort of probabilities relevant to the existence of actual causal relations—vary over time (Lewis [1980], p. 91), the probabilities (chances) appealed to in Equation (1) (and indeed throughout this paper) should be taken to be those obtaining immediately after all of the relevant interventions have occurred (cf. Lewis [1986a], p. 177).

^36^ This is not without controversy. Lewis ([1986a], p. 176) suggests that (where *A* is false) a counterfactual of the form A□→¬B (to use the symbol, □→, for the counterfactual connective adopted by Lewis ([1973b], pp. 1–2) entails A□→P(B)=0, at least where *A* and *B* concern ordinary event occurrences or non-occurrences. (Lewis’s arguments apply just as well where *A* and *B* concern the sort of variable values that we—following others in the structural equations tradition—are taking to be the relata of the actual causal relation.) Yet later he attempted to modify his counterfactual semantics to avoid this consequence (Lewis [1986b], pp. 63–5). Hawthorne ([2005]) and Williams ([2008]) argue that his proposed modification is problematic. Hájek ([unpublished]) defends the view that A□→¬B entails A□→P(B)=0, at least where *A* and *B* concern ordinary event occurrences or non-occurrences. (Hájek’s arguments also apply just as well where *A* and *B* concern variable values of the sort considered here.) I find Hájek’s arguments convincing. Still, the view that counterfactual dependence is a limiting case of probability raising (understood in terms of the inequality in Equation (1)) does not strictly require that we maintain that $\vec{X}=\vec{x}\prime □\to \neg \phi$ (in the notation employed here, $[\vec{X}=\vec{x}\prime ]\neg \phi$) entails $\vec{X}=\vec{x}\prime □\to P(\phi )=0$ (in the notation employed here, $P(\phi |do(\vec{X}=\vec{x}\prime ))=0$). All that it does require is that where $\vec{X}=\vec{x}$ and ϕ actually hold, $\vec{X}=\vec{x}□\to \phi$ and $\vec{X}=\vec{x}\prime □\to \neg \phi$ (in the notation employed here, $[\vec{X}=\vec{x}]\phi$ and $[\vec{X}=\vec{x}\prime ]\neg \phi$) are both true only if Equation (1) obtains. This seems extremely plausible in its own right.

^37^ Given his assumption of strong centring, Lewis’s closest-worlds semantics for counterfactuals implies that a counterfactual is true if its antecedent and consequent are true (Lewis [1973b], pp. 14–15, [1986a], p. 164). This implies that where $\vec{X}=\vec{x}$ and ϕ actually obtain, ‘if $\vec{X}=\vec{x}$ had obtained, then ϕ would obtain’ is true, even if $\vec{X}=\vec{x}$ didn’t actually result from an intervention or similar. The claim in the main text is that $[\vec{X}=\vec{x}]\phi$ implies $P(\phi |do(\vec{X}=\vec{x}))>0$ when these two counterfactual expressions are given a consistent semantics: if $P(\phi |do(\vec{X}=\vec{x}))>0$ is to be evaluated with respect to a world in which $\vec{X}=\vec{x}$ results from an intervention, even where actually $\vec{X}=\vec{x}$, then $[\vec{X}=\vec{x}]\phi$ should also be. Lewis’s view also implies that where actually $\vec{X}=\vec{x}$ and ϕ, ‘if $\vec{X}=\vec{x}$ had obtained, then ϕ would obtain’ is true even if ϕ actually had a chance of less than one. (This has seemed an implausible result to some; see Bennett [2003], pp. 239–41.) But Lewis wouldn’t allow that ‘if $\vec{X}=\vec{x}$ had obtained, then ϕ would have obtained’ could be true if the probability of ϕ would have been zero. After all, he maintains that nothing that has chance zero actually occurs (Lewis [1986a], pp. 175–6).

^38^ There are additional complications—to be addressed below—that arise in the probabilistic case, since probability raising under benign circumstances, though plausibly necessary, is not sufficient for actual causation. The account developed below deals with these complications by identifying a suitable generalization of Halpern and Pearl’s condition AC2(b) to the probabilistic case.

^39^ Three of the earliest philosophical discussions of the phenomenon of actual causation without probability raising are to be found in (Good [1961a], p. 318; Hesslow [1976], p. 291; Rosen [1978], pp. 607–8).

^40^ By adopting—as I have done here—a conception of probability raising that (in the manner of causal decision theory and the approaches to probabilistic causation adopted by Lewis ([1986a], pp. 175–84) and Menzies [1989], pp. 644–5, 653–7) involves counterfactual probabilities (where the counterfactuals are given a non-backtracking semantics), as opposed to conditional probabilities, we avoid cases of probability raising non-causation that arise when two events are independent effects of a third. Yet as the present example illustrates (see the main text below), the phenomenon of an event having its probability raised by a non-cause is far from being confined to such cases.

^41^ For further illustrations that this is so, see (Hitchcock [2004], pp. 410–11, 415; Menzies [1989], pp. 645–7; Edgington [1997], p. 419; Lewis [2004], pp. 79–80; Schaffer [2000], p. 41).

^42^ Indeed, what is negligible is context sensitive. In a context in which we were simply calculating approximate probability values, it was acceptable to neglect this small probability, but I take it that this is not acceptable when evaluating counterfactuals (cf. Lewis [1986a], p. 176). For a detailed argument that such probabilities cannot properly be neglected (no matter what the context) in evaluating counterfactuals, see (Hájek [unpublished], Section 5.1).

^43^ Even if we don’t accept that A□→¬B implies A□→P(B)=0 (see Footnote 36 above), it seems plausible to maintain that A□→¬B implies that if *A* obtained, then the probability of *B* would not have been large (at least where, as is the case for the counterfactual under consideration in the main text, the antecedent is false). In fact, all that is needed to establish that Halpern and Pearl’s analysis is not fully adequate to the probabilistic case is the still weaker claim that sometimes when one event is a probabilistic cause of another, it is not the case that the latter exhibits contingent counterfactual dependence upon the former (because, under the relevant contingency, the latter would have retained some degree of probability of occurring even if the former hadn’t). Even defenders of the compatibility of A□→¬B and A□→P(B)≠0 (for false *A*), such as Lewis ([1986b], pp. 63–5) and Williams ([2008], pp. 405–19), develop counterfactual semantics that make this weaker claim plausible.

^44^ I assume that A□→B is incompatible with A□→P(¬B)=0.55. One might deny this if one thinks that true-antecedent and true-consequent counterfactuals are automatically true as Lewis ([1986a], p. 164), somewhat controversially, does (see the discussion of Footnote 37, above). Indeed, the specific counterfactual presently under discussion—‘If Corleone had issued his order and Barzini had issued his order but Turk hadn’t shot, then McCluskey would have died’—is (in the scenario under consideration) a true-antecedent and true-consequent counterfactual. But this is just because the contingency that we need to appeal to in this case—that in which Barzini issues his order but Turk doesn’t shoot—happens to be actual. For reasons outlined in Footnote 24 above, it is sometimes necessary to appeal to non-actual contingencies in identifying the contingent counterfactual dependence of effect upon cause required by condition AC2(a). Where this is so, the counterfactual appealed to in AC2(b) will not be a true-antecedent and true-consequent counterfactual. And all that is needed to establish that Halpern and Pearl’s analysis is not fully adequate to the probabilistic case is that sometimes when one event is a probabilistic cause of another, it is not the case that (under the relevant contingency, which may be non-actual) the latter would have occurred if the former had (because under the relevant contingency, the latter would have retained some probability of not occurring even if the former had occurred).

^45^ One might think that, where ϕ is a primitive event or a Boolean combination of primitive events in the field generated by $V,\;P(\phi |do(\vec{V}\prime =\vec{v}\prime ))$ could be a well-defined objective chance only if $\vec{V}\prime$ includes enough variables. For example, one might think that $\vec{V}\prime$ must include all exogenous variables $\vec{U}$, so that $\vec{V}\prime =\vec{v}\prime$ incorporates a complete specification of the context $\vec{U}=\vec{u}$, if $P(\phi |do(\vec{V}\prime =\vec{v}\prime ))$ is to express a well-defined objective chance for ϕ. (Though Hitchcock ([unpublished], Section 14) suggests that, under certain conditions, specifying the values of certain sets of variables that do not include all of the variables in $\vec{U}$ may also yield a chance value for ϕ.) Moreover, one might think that the set of exogenous variables must be fairly rich if $P(\phi |do(\vec{U}=\vec{u}))$ is to express an objective chance (for all Boolean combinations ϕ of primitive events in the field generated by the variables in V and for all possible values $\vec{u}$ of $\vec{U}$). One might thus think that it ought to be taken as a requirement of model appropriateness that the set of exogenous variables be rich enough to generate such chances.

^46^ Like Halpern and Pearl’s AC, PC relativizes the notion of actual causation to a model (in this case, a probabilistic model). If one takes model-relativity to be an objectionable feature, then one could avoid it by saying that $\vec{X}=\vec{x}$ is an actual cause of ϕ*simpliciter*, provided that there exists at least one appropriate probabilistic causal model relative to which PC is satisfied (cf. Section 4, above). Most of the criteria for an appropriate deterministic SEM that have been advanced in the literature (see Hitchcock [2001a], p. 287; Halpern and Hitchcock [2010], pp. 394–9; Blanchard and Schaffer [forthcoming], Section 1) apply just as well to probabilistic causal models.

^47^ PC could be stated more simply than it is in the main text. This is because condition PC2(a) is, in fact, redundant given PC2(b). The inequality appealed to in PC2(b) is required to hold for all subsets $\vec{Z}\prime$ of $\vec{Z}$. Where $\vec{Z}\prime =Ø$, this inequality is identical to the one that is appealed to in PC2(a). Despite the possibility of simplification, the version of PC stated in the main text is in a way more perspicuous because it makes clear the formal analogy between **AC** and PC, with PC2(a) isolating the contingent probabilistic dependence requirement made by PC just as AC2(a) constitutes the contingent counterfactual dependence requirement made by AC. Isolating the contingent probabilistic dependence requirement in condition PC2(a) also helps lend clarity to the discussion below of how PC handles the probabilistic preemption scenario, as well as other interesting probabilistic causal scenarios. Thanks to an anonymous referee for pressing me to say more about why PC is stated in its present form.

^48^ If—as was suggested in the discussion of Section 6—counterfactual dependence is taken to be a limiting case of probabilistic dependence (when probabilistic dependence is understood, as it is here, in terms of counterfactuals about probabilities), it is plausible that PC and **AC** yield equivalent verdicts in deterministic causal scenarios, where all probabilities are ones or zeros.

^49^ I mentioned in Footnote 18 that the version of Halpern and Pearl’s condition AC2(b), given in (Halpern and Pearl [2001]) and stated in Section 4, above, has a probabilistic analogue—namely, my PC2(b)—that is superior in its handling of probabilistic preemption to the obvious probabilistic analogue of the somewhat different version of AC2(b) given in (Halpern and Pearl [2005]). Though I won’t go into the details (readers familiar with Halpern and Pearl’s ([2005]) account should be able to surmise them for themselves), the obvious probabilistic analogue of the later version of AC2(b) would allow that Corleone’s action was a cause only if P(D=1|do(C=1&B=1&S=1))>P(D=1|do(C=0&B=1)). But this inequality does not hold in the present case, since P(D=1|do(C=1&B=1&S=1))≈0.5, while P(D=1|do(C=0&B=1))≈0.81. Since Corleone’s action *is* a cause, PC2(b) yields the correct result, while the obvious probabilistic analogue of the later version of AC2(b) does not. So PC2(b) is the correct condition. As I also noted in Footnote 18, insofar as we are interested in developing a unified account of deterministic and probabilistic causation, this would appear to give us an additional reason for preferring the original version of AC2(b) in the deterministic case.

^50^ In what follows, I shall leave this parenthetical qualification implicit in all cases where $\vec{W}$ is empty.

^51^ Following Hitchcock ([2004], pp. 404–5), I also think that acknowledging the contrastive nature of probabilistic causation is the correct way to deal with a well-known example of probabilistic causation due to Rosen ([1978], pp. 607–8) and the similar examples that are described in (Salmon [1984], pp. 194–201). Though, for reasons of space, I won’t demonstrate how the revised PC handles those examples here, the interested reader should have little trouble seeing how it does so, particularly in light of the discussion of Hitchcock ([2004], pp. 404–5).

^52^ Of course, both in this case and in the medical case above, there are more than two possible contrast sets that might be specified. Other contrast sets include {S=25,S=75} in the driving example and {M=0,M=1} in the medical example. (The reader can verify that relative to these contrast sets, the revised PC yields negative verdicts about actual causation in the two cases.) So causal claims that fail to specify a contrast set are in fact multiply ambiguous in these cases. In discussing the examples, I just picked out two particularly interesting contrast sets.

^53^ We might also require that for every ϕ′∈ϕ′, there is some event of the form $\vec{X}=\vec{x}\prime$ in $\vec{X}=\vec{x}\prime$ such that (i) and (ii) hold with respect to precisely this $\vec{X}=\vec{x}\prime$ and this ϕ′ (cf. Schaffer [2005], p. 348).

^54^ For simplicity, here and in what follows, I drop reference to contrast sets where no ambiguity will result.

^55^ In fact, a further adjustment to PC would be needed to capture (e). Specifically, one would have to limit the subsets $\vec{Z}\prime$ of $\vec{Z}$ appealed to in PC2(b) to those subsets that don’t include variables that figure in ϕ. I will leave this qualification implicit from now on.

^56^ Although I take (e) to capture one notion of negative causal relevance, I don’t think that there is an analogous sense in which positive causal relevance is used. That is, I don’t think that we would ever take ‘$\vec{X}=\vec{x}$ is positively causally relevant to ϕ’ to mean (e′) ‘$\vec{X}=\vec{x}$ raises the probability of ϕ along a causal pathway representing a process that is complete, except ¬ϕ holds’. (Though we might mean (c)—see the main text above—which is similar.)

^57^ Note that on some disambiguations of these notions (for example, (c) and (c′)), $\vec{X}=\vec{x}$ being positively causally relevant to ϕ isn’t incompatible with $\vec{X}=\vec{x}$ also being negatively causally relevant to ϕ (cf. Hitchcock [2001b], p. 370). By contrast, ‘$\vec{X}=\vec{x}$ caused ϕ’ and ‘$\vec{X}=\vec{x}$ prevented ϕ’ are incompatible (because the former implies that ϕ holds, whereas the latter implies that ϕ doesn’t hold).

^58^ Since nothing in the following discussion of Twardy and Korb’s account turns on issues of contrastivity, I compare their account with the original (non-contrastive) definitions AC and PC for simplicity.

^59^ As usual, it is important that the foregoing counterfactuals are evaluated with respect to worlds in which the variables—in particular, *T*—have the specified values as a result of (hard) interventions, or the like. Again, this is what the do(·) operator indicates.

^60^ In the original example, it was (for calculational simplicity) stated that the chance of such an event was ‘negligible’, but not that it was zero. Furthermore, we could stipulate a non-negligible probability of such an event without changing the basic structure of the example.

^61^ Indeed, even if I am mistaken about how they view the relation between causal relevance and actual causation, this example appears to create problems for them, since their account yields an asymmetry in the causal status of *C* = 1 and *B* = 1 with respect to *D* = 1 in this case; yet it seems that either both or neither should count as causally relevant to *D* = 1.

^62^ This fact also underlies the rather counterintuitive verdict that Twardy and Korb’s account yields concerning the ‘stochastic assassin’ case that they discuss (Twardy and Korb [2011], pp. 909–11). Though I shall not show it here, the interested reader can verify that the analysis that I have proposed (namely, PC), unlike Twardy and Korb’s analysis, yields the expected result about the causal status of the event ‘supervisor’s aiming’ in that example.

## References

[axv056-B1] Baumgartner M. [2013]: ‘A Regularity Theoretic Approach to Actual Causation’, Erkenntnis*,* 78, pp. 85–109.

[axv056-B2] Bennett J. [2003]: A Philosophical Guide to Conditionals*,* Oxford: Oxford University Press.

[axv056-B3] Blanchard T., Schaffer J. [forthcoming]: ‘Cause without Default’, in Beebee H., Hitchcock C., Price H. (*eds*), Making a Difference*,* Oxford: Oxford University Press.

[axv056-B4] Edgington D. [1997]: ‘Mellor on Chance and Causation’, British Journal for the Philosophy of Science*,* 48, pp. 411–33.

[axv056-B5] Eells E. [1991]: Probabilistic Causality*,* Cambridge: Cambridge University Press.

[axv056-B6] Emery N. [2015]: ‘Chance, Possibility, and Explanation’, British Journal for the Philosophy of Science*,* 66, pp. 95–120.

[axv056-B7] Frigg R., Hoefer C. [2010]: ‘Determinism and Chance from a Humean Perspective’, in Dieks D., Gonzalez W., Hartmann S., Weber M., Stadler F., Uebel T. (*eds*), The Present Situation in the Philosophy of Science*,* Berlin and New York: Springer, pp. 351–71.

[axv056-B8] Frigg R., Hoefer C. [2015]: ‘The Best Humean System for Statistical Mechanics’, Erkenntnis, 80, pp. 551–74.

[axv056-B9] Frisch M. [2014]: ‘Why Physics Can’t Explain Everything’, in Wilson A. (*ed.*), Chance and Temporal Asymmetry, Oxford: Oxford University Press.

[axv056-B10] Glynn L. [2009]: A Probabilistic Analysis of Causation*,* PhD thesis, University of Oxford.

[axv056-B11] Glynn L. [2010]: ‘Deterministic Chance’, British Journal for the Philosophy of Science*,* 61, pp. 51–80.

[axv056-B12] Glynn L. [2011]: ‘A Probabilistic Analysis of Causation’, British Journal for the Philosophy of Science*,* 62, pp. 343–92.

[axv056-B13] Glynn L. [2013]: ‘Of Miracles and Interventions’, Erkenntnis*,* 78, pp. 43–64.

[axv056-B14] Godszmidt M., Pearl J. [1992]: ‘Rank-Based Systems: A Simple Approach to Belief Revision, Belief Update, and Reasoning about Evidence and Actions’, in B. Nebel, C. Rich, and W. Swartout (*eds*), Proceedings of the Third International Conference on Knowledge Representation and Reasoning*,* San Mateo, CA: Morgan Kaufmann, pp. 661–72.

[axv056-B15] Good I. J. [1961a]: ‘A Causal Calculus (I)’, British Journal for the Philosophy of Science*,* 11, pp. 305–18.

[axv056-B16] Good I. J. [1961b]: ‘A Causal Calculus (II)’, British Journal for the Philosophy of Science*,* 12, pp. 43–51.

[axv056-B17] Hájek A. [unpublished]: ‘Most Counterfactuals Are False’, available at <http://philosophy.anu.edu.au/sites/default/files/Most%20counterfactuals%20are%20false.1.11.11_0.pdf>.

[axv056-B18] Halpern J. Y. [2008]: ‘Defaults and Normality in Causal Structures’, in Brewka G., Lang J. (*eds*), Proceedings of the Eleventh International Conference on Principles of Knowledge Representation and Reasoning*,* Menlo Park, CA: AAAI Press, pp. 198–208.

[axv056-B19] Halpern J. Y. [unpublished]: ‘Appropriate Causal Models and Stability of Causation’, available at <www.cs.cornell.edu/home/halpern/papers/causalmodeling.pdf>.

[axv056-B20] Halpern J. Y., Hitchcock C. [2010]: ‘Actual Causation and the Art of Modeling’, in Dechter R., Geffner H., Halpern J. Y. (*eds*), Heuristics, Probability and Causality: A Tribute to Judea Pearl*,* London: College Publications, pp. 383–406.

[axv056-B21] Halpern J. Y., Hitchcock C. [2015]: ‘Graded Causation and Defaults’, British Journal for the Philosophy of Science, 66, pp. 413–57.

[axv056-B22] Halpern J. Y., Pearl J. [2001]: ‘Causes and Explanations: A Structural-Model Approach, Part I: Causes’, in J. S. Breese and D. Koller (*eds*), Proceedings of the Seventeenth Conference on Uncertainty in Artificial Intelligence*,* San Francisco, CA: Morgan Kaufmann, pp. 194–202.

[axv056-B23] Halpern J. Y., Pearl J. [2005]: ‘Causes and Explanations: A Structural-Model Approach, Part I: Causes’, British Journal for the Philosophy of Science, 56, pp. 843–87.

[axv056-B24] Handfield T., Twardy C. R., Korb K. B., Oppy G. [2008]: ‘The Metaphysics of Causal Models: Where’s the Biff?’, Erkenntnis*,* 68, pp. 149–68.

[axv056-B25] Hawthorne J. [2005]: ‘Chance and Counterfactuals’, Philosophy and Phenomenological Research*,* 70, pp. 396–405.

[axv056-B26] Hesslow G. [1976]: ‘Two Notes on the Probabilistic Approach to Causality’, Philosophy of Science*,* 43, pp. 290–2.

[axv056-B27] Hitchcock C. [1996a]: ‘Farewell to Binary Causation’, Canadian Journal of Philosophy*,* 26, pp. 267–82.

[axv056-B28] Hitchcock C. [1996b]: ‘The Role of Contrast in Causal and Explanatory Claims’, Synthese*,* 107, pp. 395–419.

[axv056-B29] Hitchcock C. [2001a]: ‘The Intransitivity of Causation Revealed in Equations and Graphs’, Journal of Philosophy*,* 98, pp. 194–202.

[axv056-B30] Hitchcock C. [2001b]: ‘A Tale of Two Effects’, Philosophical Review*,* 110, pp. 361–96.

[axv056-B31] Hitchcock C. [2004]: ‘Do All and Only Causes Raise the Probabilities of Effects?’, in Collins J., Hall N., Paul L. (*eds*), Causation and Counterfactuals*,* Cambridge, MA: MIT Press, pp. 403–17.

[axv056-B32] Hitchcock C. [2007]: ‘Prevention, Preemption, and the Principle of Sufficient Reason’, Philosophical Review*,* 116, pp. 495–532.

[axv056-B33] Hitchcock C. [2009]: ‘Causal Modelling’, in Beebee H., Hitchcock C., Menzies P. (*eds*), The Oxford Handbook of Causation*,* New York: Oxford University Press, pp. 299–314.

[axv056-B34] Hitchcock C. [unpublished]: ‘Cause and Chance’, available at <www.uni-konstanz.de/philosophie/fe/files/christopher_hitchcock_1.pdf>.

[axv056-B35] Hoefer C. [2007]: ‘The Third Way on Objective Probability: A Sceptic’s Guide to Objective Chance’, Mind*,* 116, pp. 549–96.

[axv056-B36] Hopkins M., Pearl J. [2003]: ‘Clarifying the Usage of Structural Models for Common-Sense Causal Reasoning’, in Proceedings of the AAAI Spring Symposium on Logical Formalizations of Commonsense Reasoning*,* Menlo Park, CA: AAAI Press, pp. 83–9.

[axv056-B37] Ismael J. [2009]: ‘Probability in Deterministic Physics’, Journal of Philosophy*,* 106, pp. 89–108.

[axv056-B38] Ismael J. [2012]: ‘A Modest Proposal about Chance’, Journal of Philosophy*,* 108, pp. 416–42.

[axv056-B39] Kvart I. [2004]: ‘Causation: Probabilistic and Counterfactual Analyses’, in Causation and Counterfactuals*,* Cambridge, MA: MIT Press, pp. 359–86.

[axv056-B40] Lewis D. [1973a]: ‘Causation’, Journal of Philosophy*,* 70, pp. 556–67.

[axv056-B41] Lewis D. [1973b]: Counterfactuals*,* Oxford: Oxford University Press.

[axv056-B42] Lewis D. [1979]: ‘Counterfactual Dependence and Time’s Arrow’, Noûs*,* 13, pp. 455–76.

[axv056-B43] Lewis D. [1980]: ‘A Subjectivist’s Guide to Objective Chance’, in Jeffrey R. (*ed.*), Studies in Logic and Inductive Probability*,* Volume 2, Berkeley: University of California Press, pp. 267–97.

[axv056-B44] Lewis D. [1986a]: ‘Postscripts to “Causation”’, in his Philosophical Papers*,* Volume 2, Oxford: Oxford University Press, pp. 172–213.

[axv056-B45] Lewis D. [1986b]: ‘Postscripts to “Counterfactual Dependence and Time’s Arrow”’, in his Philosophical Papers*,* Volume 2, Oxford: Oxford University Press, pp. 52–66.

[axv056-B46] Lewis D. [2004]: ‘Causation as Influence’, in Collins J., Hall N., Paul L. (*eds*), Causation and Counterfactuals*,* Cambridge, MA: MIT Press, pp. 75–106.

[axv056-B47] List C., Pivato M. [2015]: ‘Emergent Chance’, Philosophical Review*,* 124, pp. 119–152.

[axv056-B48] Loewer B. [2001]: ‘Determinism and Chance’, Studies in History and Philosophy of Modern Physics*,* 32, pp. 609–20.

[axv056-B49] Mackie J. [1965]: ‘Causes and Conditions’, American Philosophical Quarterly*,* 2, pp. 245–64.

[axv056-B50] Menzies P. [1989]: ‘Probabilistic Causation and Causal Processes: A Critique of Lewis’, Philosophy of Science*,* 56, pp. 642–63.

[axv056-B51] Menzies P. [1996]: ‘Probabilistic Causation and the Pre-emption Problem’, Mind*,* 105, pp. 85–117.

[axv056-B52] Paul L. A., Hall N. [2013]: Causation: A User’s Guide*,* Oxford: Oxford University Press.

[axv056-B53] Pearl J. [1995]: ‘Causal Diagrams for Empirical Research’, Biometrika*,* 82, pp. 669–710.

[axv056-B54] Pearl J. [2000]: Causality: Models, Reasoning, and Inference*,* Cambridge: Cambridge University Press.

[axv056-B55] Pearl J. [2009]: Causality: Models, Reasoning, and Inference*,* Cambridge: Cambridge University Press.

[axv056-B56] Reichenbach H. [1971]: The Direction of Time, Mineola, NY: Dover Publications.

[axv056-B57] Rosen D. [1978]: ‘In Defense of a Probabilistic Theory of Causality’, Philosophy of Science*,* 45, pp. 604–13.

[axv056-B58] Salmon W. [1984]: Scientific Explanation and the Causal Structure of the World*,* Princeton, NJ: Princeton University Press.

[axv056-B59] Schaffer J. [2000]: ‘Overlappings: Probability-Raising without Causation’, Australasian Journal of Philosophy*,* 78, pp. 40–6.

[axv056-B60] Schaffer J. [2001]: ‘Causes as Probability Raisers of Processes’, Journal of Philosophy*,* 98, pp. 75–92.

[axv056-B61] Schaffer J. [2005]: ‘Contrastive Causation’, Philosophical Review*,* 114, pp. 327–58.

[axv056-B62] Schaffer J. [2013]: ‘Causal Contextualisms’, in Blaauw M. (*ed.*), Contrastivism in Philosophy*,* New York: Routledge, pp. 35–63.

[axv056-B63] Spirtes P., Glymour C., Scheines R. [2000]: Causation, Prediction, and Search*,* Cambridge, MA: MIT Press.

[axv056-B64] Strevens M. [2007]: ‘Review of Woodward, *Making Things Happen*’, Philosophy and Phenomenological Research*,* 74, pp. 233–49.

[axv056-B65] Strevens M. [2008]: ‘Comments on Woodward, *Making Things Happen*’, Philosophy and Phenomenological Research*,* 77, pp. 171–92.

[axv056-B66] Suppes P. [1970]: A Probabilistic Theory of Causality*,* Amsterdam: North-Holland.

[axv056-B67] Twardy C. R., Korb K. B. [2011]: ‘Actual Causation by Probabilistic Active Paths’, Philosophy of Science*,* 78, pp. 900–13.

[axv056-B68] Twardy C. R., Korb K. B. [unpublished]: ‘Actual Causation by Probabilistic Active Paths (Supplement)’, 〈http://philsci-archive.pitt.edu/8878/〉

[axv056-B69] Weslake B. [forthcoming]: ‘A Partial Theory of Actual Causation’, British Journal for the Philosophy of Science.

[axv056-B70] Williams J. R. G. [2008]: ‘Chances, Counterfactuals, and Similarity’, Philosophy and Phenomenological Research*,* 77, pp. 385–420.

[axv056-B71] Woodward J. [2005]: Making Things Happen: A Theory of Causal Explanation*,* Oxford: Oxford University Press.

[axv056-B72] Woodward J. [2008]: ‘Response to Strevens’, Philosophy and Phenomenological Research*,* 77, pp. 193–212.

